# Developing a Quick Isolation Bed Inquiry System During the COVID-19 Outbreak: User-Centered Design Approach Based on the Toyota Production System

**DOI:** 10.2196/67152

**Published:** 2025-10-17

**Authors:** Chien-Chung Lin, Jian-Hong Shen, Shan-Li Chang, Tai-Chih Kuo, Hui-Ling Huang, Cheng-Yi Peter Lin, Hung-Meng Huang

**Affiliations:** 1 Department of Surgery and Orthopedic Surgery Taipei City Hospital University of Taipei Taipei City Taiwan; 2 Department of Finance Chihlee University of Technology New Taipei City Taiwan; 3 Department of Energy and Refrigerating Air-Conditioning Engineering National Taipei University of Technology Taipei City Taiwan; 4 Department of Biochemistry & Molecular Cell Biology School of Medicine Taipei Medical University Taipei Taiwan; 5 Department of Orthopedic Surgery Taipei City Hospital Taipei City Taiwan; 6 Institute of Hospital & Health Care Administration National Yang Ming Chiao Tung University Taipei City Taiwan; 7 Department of Otorhinolaryngology Taipei City Hospital Taipei City Taiwan

**Keywords:** autonomation, continuous improvement, information technology, quality improvement, health care IT, just-in-time, lean, problem solving, PDCA, Toyota business practice, Toyota production system, value stream map

## Abstract

**Background:**

During the COVID-19 outbreak in May 2021, our hospital—designated as a specialized facility for severely infected patients—faced critical staff and resource shortages. The urgent need for efficient bed management to ensure timely admissions underscored the inefficiencies of the manual, phone-based allocation process, which averaged 454 seconds per query. Traditional IT solutions were not feasible due to time and cost constraints.

**Objective:**

This study aims to design and implement a rapid, zero-cost Quick Isolation Bed Inquiry System that provides real-time bed information and enables timely admissions without requiring additional workforce or expense.

**Methods:**

We conducted a 3-cycle pre-post quality improvement study guided by the Toyota business practice (TBP), an 8-step problem-solving framework. After clarifying the problem and constructing a value stream map, we identified bottlenecks. A user-centered solution was developed by leveraging an underutilized data export function in the hospital’s bed inquiry platform. Using Microsoft Excel Visual Basic for Applications, we automated the filtering and display of relevant bed information. The primary outcomes were process time and number of steps; secondary outcomes included staff time savings and system accuracy.

**Results:**

The baseline manual process required 25 steps and 454 seconds to complete a query. The new system reduced this to 3 steps and 12 seconds, representing a 97.4% gain in efficiency. Single-click execution generated 3 outputs (administrative PDF, large-screen display, and mobile version) in 4 seconds, with distribution to the hospital LINE group completed in 7 seconds. Reliability reached 100%, with continuous availability through virtual private network access. Development and debugging were completed within 3 days using only existing resources. Postpandemic, the system was adapted for general ward management with minimal modifications.

**Conclusions:**

Applying TBP enabled the rapid development of a user-centered, zero-cost bed management tool by repurposing existing digital assets. The intervention markedly improved efficiency, reliability, and usability without additional staffing or expenditure, providing a scalable model for agile health care systems operating under resource constraints. Future work will focus on deeper automation, such as application programming interface–based real-time updates, and on evaluating downstream impacts on patient flow and bed turnover.

## Introduction

### Problem Description

As of October 2020, over 37 million people worldwide had been diagnosed with COVID-19, and more than 1 million had died, reflecting a case-fatality rate of about 2.9% [1]. The pandemic’s effects were especially severe in the United States, which accounted for roughly one-fifth of global infections and deaths [1]. It also exposed major weaknesses in hospital bed-management systems and highlighted that timely patient allocation was crucial for survival during surge periods [2,3].

This global crisis manifested locally in Taiwan in May 2021, when the country experienced a severe COVID-19 outbreak. In Taipei City, cases rose sharply from 27 in April to 2745 in May [4]. Our hospital, Taipei City Hospital Heping Branch, was designated as the capital region’s primary COVID-19 treatment facility, managing 148 isolation beds for patients with COVID-19. This designation created an immediate surge in admission demand while the hospital simultaneously faced critical staffing and resource shortages.

The most urgent challenge was managing bed availability and rapidly assigning beds to patients, as mandated by the National Disease Control Bureau. Under normal conditions, bed allocation was managed manually by the Medical Affairs Office. The pandemic, however, exposed major inefficiencies in this approach. We lacked both sufficient personnel and a clear system to provide real-time updates on bed availability to hospital management. Bed control physicians, responsible for assignments, had to rely on a manual, time-consuming process of calling each ward individually to check availability. This method was inefficient and risked compromising patient safety and treatment quality due to delays.

### Existing Knowledge

A comprehensive literature review revealed limited research on rapid bed allocation methods or real-time bed assignment platforms, particularly in emergency contexts [5-11]. The identified research gap included both the absence of rapid implementation frameworks for bed management systems during health care crises and the lack of real-time visibility tools capable of functioning under staffing shortages and high-stress conditions. Although commercial electronic bed management systems offer features such as real-time monitoring, predictive analytics, and integration capabilities [12-18], these typically require long implementation timelines and substantial resource investments, making them unsuitable for emergency deployment.

This gap reflects a broader challenge in health care IT: how to rapidly develop and deploy digital solutions during crisis conditions when traditional development cycles are not feasible [19-21]. The research question guiding this study is as follows: “Can structured problem-solving methodologies from manufacturing be effectively adapted to develop health care IT solutions under extreme time and resource constraints?”

### Rationale

With no dedicated personnel or prior models to follow, a solution was required that could be developed rapidly using existing resources. In the absence of alternative theoretical frameworks in the scientific literature for rapid health care IT implementation during crises, we turned to the Toyota production system (TPS). We selected the Toyota business practice (TBP) framework of the TPS—a systematic 8-step problem-solving methodology—for 3 evidence-based reasons: (1) demonstrated success in health care quality improvement across various processes [22-27]; (2) emphasis on leveraging existing resources rather than requiring new investments [27-36]; and (3) proven applicability in our institution’s previous digital health projects [30].

### Objective

Therefore, the objective of this study is to provide a detailed case report on applying the structured 8-step TBP framework to rapidly design, develop, and implement a low-cost, user-centered bed inquiry system using only existing hospital resources. This study documents the iterative process of problem identification, root cause analysis, and solution development, offering a practical model for agile problem solving in health care crises.

## Methods

### Study Design

This quality improvement implementation study employed a pre-post design using TBP’s structured problem-solving methodology across 3 iterative cycles. The study was conducted in accordance with implementation research principles and the SQUIRE (Standards for Quality Improvement Reporting Excellence) 2.0 guidelines, with particular emphasis on real-world effectiveness and the timely deployment of solutions [37-39].

### Setting and Context

The study was conducted at Taipei City Hospital Heping Branch, a 411-bed public hospital designated as the primary COVID-19 treatment facility for Taipei City. During the study period, the hospital managed 148 isolation beds across 4 wards (A9, A8, A7, and A6), with complex occupancy rules requiring gender-matched double rooms.

### Methodological Framework: The Toyota Business Practice

The study was structured around the TBP, a formal 8-step problem-solving methodology derived from the core principles of the TPS [31-35]. This framework, also widely referred to in TPS literature as the A3 problem-solving process, provides a systematic and disciplined approach to identifying, analyzing, and resolving operational challenges [35,40]. The 8 steps are (1) clarify the problem, (2) break down the problem, (3) set a target, (4) analyze the root cause, (5) develop countermeasures, (6) implement countermeasures, (7) evaluate results and processes, and (8) standardize and share the successful approach [27,31,35].

**Figure 1:**
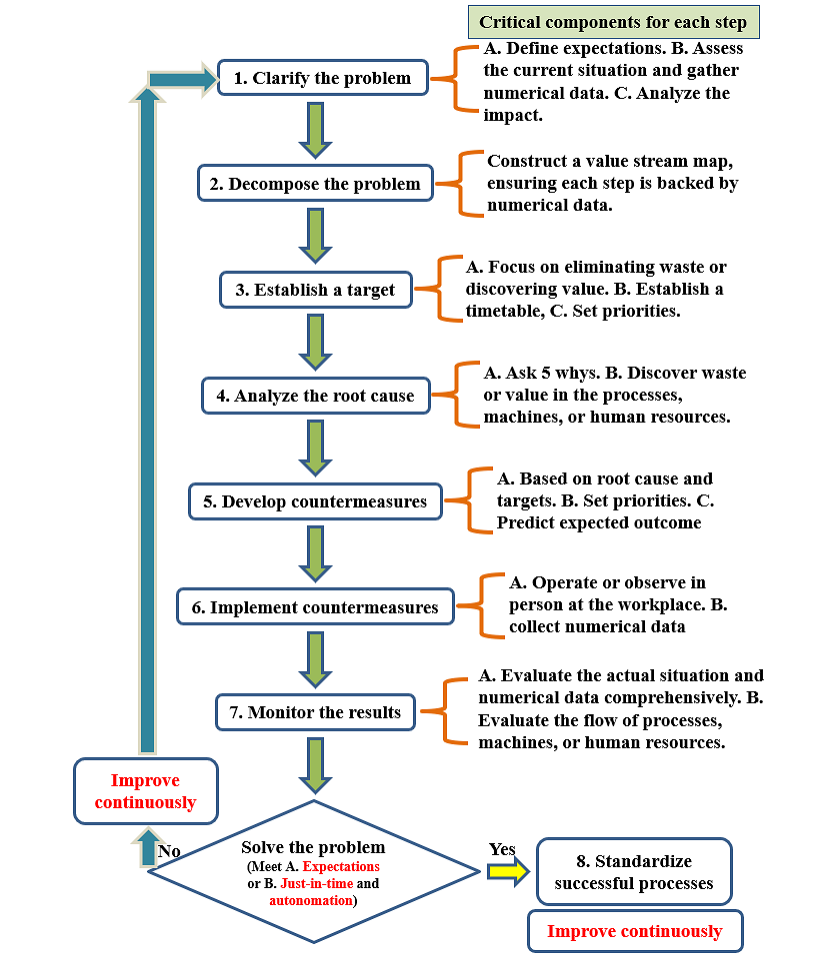
Overview of the Toyota business practice problem-solving process, highlighting the key components of each step.

Figure 1 outlines the TBP problem-solving process, highlighting the key elements at each stage.

Ohno [32] consistently emphasized his strong belief in the concept of *gemba* (on-site work). Even after joining Toyota’s top management, he continued to spend most of his time on the shop floor [32]. Consequently, it is fair to say that TPS is fundamentally centered on on-site management [32-35]. According to Ohno, work activities are classified based on their contribution to value into 3 categories: waste, non-value–added work (necessary but non-value–adding activities), and value-added work [32,33]. Waste refers to the use of resources without creating value. Non-value–added work, although necessary under current circumstances, does not generate value and is still considered a form of waste. Value-added work encompasses activities that directly enhance the original value [32,33]. TPS principles assert that a product or service is valuable only if it meets the needs and expectations of the user or customer [32-35]. Careful on-site observation is essential to clearly distinguish value-adding activities from wasteful actions.

To situate this methodology within the broader context of quality improvement science, it is important to recognize its structural alignment with the plan-do-check-act (PDCA) cycle, a foundational model for continuous improvement [35,40,41]. The TBP framework maps directly onto the PDCA cycle, demonstrating that it is a rigorous, cyclical quality improvement methodology rather than an ad hoc process [35,40]. The *plan* phase of PDCA is addressed by the first 5 steps of TBP, which emphasize deep problem understanding and planning. The *do, check,* and *act* phases correspond directly to TBP steps 6, 7, and 8, respectively. This alignment, highlighting the critical importance of the planning phase, reinforces TBP as a powerful tool for effective issue resolution. The focus on continuous improvement and the cyclical nature of TBP mirrors that of PDCA, enhancing the methodology’s credibility and generalizability [35,40,41].

### An Iterative, User-Centered Design Process

While TBP provided the overarching structure, the study’s execution closely followed the principles of user-centered design [32,33,42,43]. User-centered design is a design philosophy and process that places the end user at the center of every development stage, focusing on their needs, workflows, and context to create tools that are not only functional but also highly usable and effective [42]. The 3 TBP cycles described in this paper can be formally interpreted as sequential phases of a comprehensive user-centered design process [32,33,42].

The study unfolded through the following user-centered design phases.

### The First Cycle of TBP: Defining User Needs and Workflow Analysis

#### Step 1: Clarify the Problem

The initial cycle focused on empathy and user research, a cornerstone of user-centered design [42,43]. It involved direct on-site observation and collaboration with the primary users—the bed control physicians.

##### TPS Thinking 1

Problem identification begins with a need or expectation [32]. Once expectations are defined, direct observation and data collection at the workplace are essential to assess the current situation. A problem is identified when a gap exists between expectations and the current state [32,33].

##### Expectations

The hospital superintendent required immediate and accurate information on bed availability to ensure the prompt admission of patients with COVID-19. Bed control physicians—including the Vice Superintendent, Head of Internal Medicine, and Head of Surgery—were responsible for posting available bed information in the Critical Care Bed Coordination Group via the LINE messaging app (LY Corp) and for promptly assigning beds to patients.

##### Current Situation

After clarifying leadership’s expectations, we followed TPS principles by conducting on-site observations and collecting real-time data, rather than relying on secondhand information or meetings. This direct assessment revealed that existing hospital procedures and systems were unable to provide timely bed availability information, highlighting a clear gap between expectations and actual performance.

##### Impact

Delays in bed availability information could compromise patient safety and treatment quality, making this a critical issue that must be addressed.

#### Step 2: Break Down the Problem

##### TPS Thinking 2

To break down the problem, it is essential to visit the workplace—either to perform tasks firsthand or to observe directly—while collecting relevant numerical data. This information is then used to create a value stream map (VSM) to visualize the problem [32,33,36].

##### Manual Process for Critical Care Bed Coordination

Initially, our hospital opened 2 wards (A9 and A8). Under emergency conditions, bed control physicians had to call each ward individually to check bed availability. As patient numbers increased, 3 wards (A9, A8, and A7) were opened within a week, eventually expanding to 4 wards (A9, A8, A7, and A6) with a total of 148 beds. Bed control physicians contacted nursing staff or clerks in each ward to count available beds, recorded the data, converted it into text, and posted it in the Critical Care Bed Coordination Group on the LINE messaging app.

It is important to note that hospital policy requires patients of the same gender (except couples) to share a double room in isolation wards. Therefore, when 1 bed in a double room is occupied, the patient’s gender must be identified and recorded to ensure compliance when displaying the remaining vacant bed.

**Figure 2:**
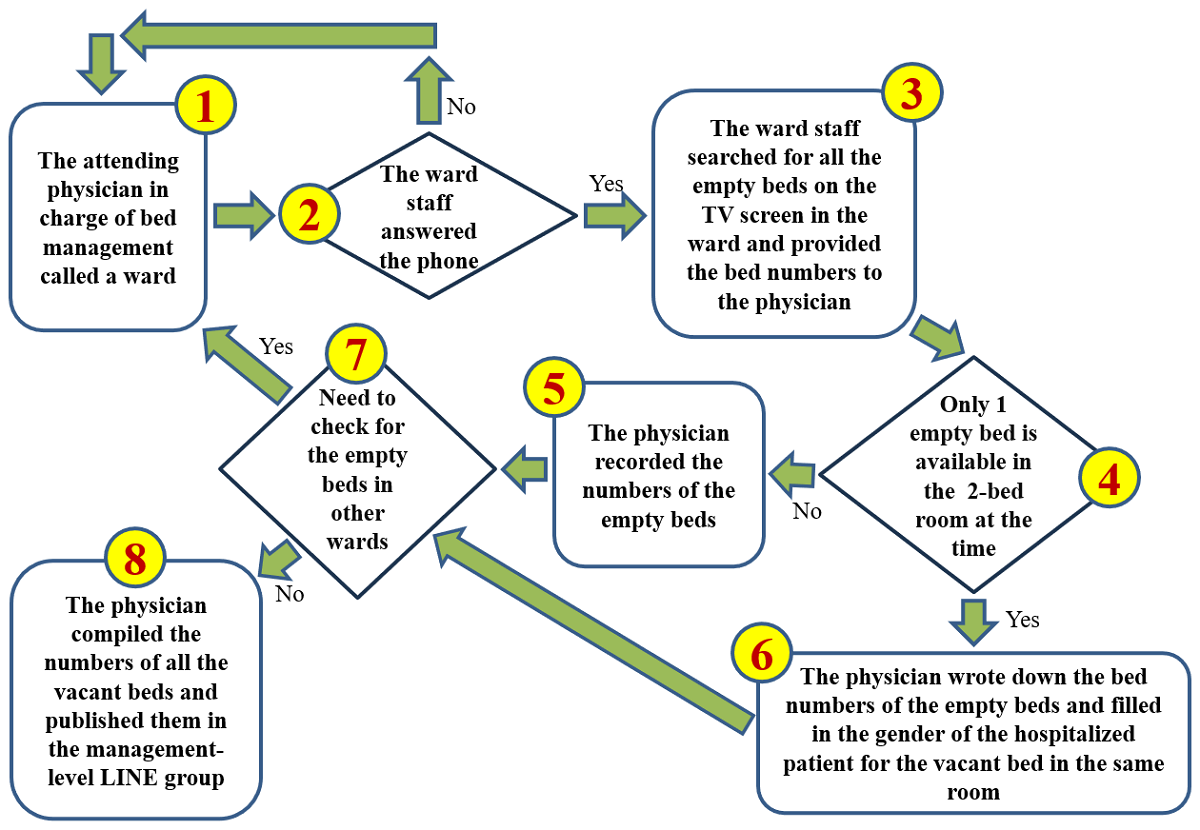
Mapping the entire process of querying bed availability resulted in the creation of a value stream map.

As we had no prior experience or information on querying bed availability, we conducted the search ourselves. Figure 2 illustrates that each step in the bed availability query process provided valuable insights for bed control physicians. Through hands-on operation and data collection, we gathered key numerical data, summarized in Multimedia Appendix 1. This table highlights the shortest time required to retrieve available bed information, as observed by one author acting (C-CL) as a bed control physician. To address uncertainties in step 2, such as unanswered calls, we excluded non-value–added activities, including calls going unanswered, wait times exceeding 30 seconds, or the need to redial. Consequently, the data collected include only instances in which ward staff answered the call within 30 seconds. We also recorded the time required for ward staff to locate available beds on the TV screen and for the surgeon to document the vacant bed numbers.

Multimedia Appendix 1 shows that the average shortest query time was 454 seconds (7 minutes and 34 seconds), which fell short of management’s expectations for immediate bed availability to expedite patient assignments. To improve bed management, the hospital superintendent mandated that bed control physicians report bed availability to the Critical Care Bed Coordination Group at 8 AM, noon, 4 PM, and 8 PM daily. To comply with this directive, physicians had to begin checking available beds 20-30 minutes before each reporting time.

Our analysis, following step 1 (clarify the problem) and step 2 (break down the problem), clearly revealed why “current hospital procedures and systems were unable to provide timely bed availability information” relative to “leadership’s expectations.” Specifically, the hospital superintendent expected immediate and accurate information on bed availability to ensure the prompt admission of patients with COVID-19. Bed control physicians were responsible for posting available bed information in the Critical Care Bed Coordination Group via the LINE messaging app and for promptly assigning beds.

However, our on-site observations revealed a significant gap. The “current hospital procedures and systems were unable to provide timely bed availability information,” primarily due to a manual, labor-intensive process involving the following steps: (1) Bed control physicians individually called each ward to check availability. (2) Ward staff manually searched for empty beds on their TV screens and verbally relayed the information. (3) Physicians recorded this information, converted it into text, and manually posted it. (4) For double rooms, the gender of existing patients had to be identified and recorded to ensure policy compliance for new admissions.

The extreme inefficiency of this manual process is highlighted in Multimedia Appendix 1, which shows that the average shortest query time for bed availability was 454 seconds (7 minutes and 34 seconds), even under ideal conditions (calls answered within 30 seconds). This fell far short of the “immediate” information expected by management. Moreover, the hospital superintendent mandated reports at 8 AM, noon, 4 PM, and 8 PM daily, requiring physicians to begin checking beds 20-30 minutes before each reporting time. This substantial preparation time underscores how the manual system failed to meet leadership’s expectations for rapid, real-time updates. The process was not only slow but also prone to delays due to unanswered calls and staff busyness.

Attentive readers may note that many of our process evaluations were conducted using “best-case” or “shortest time” scenarios. Multimedia Appendix 2 clarifies our methodology and explains the rationale for adopting these scenarios.

#### Step 3: Establish a Target

##### TPS Thinking 3

All problems arise from the existence of waste. Prioritize eliminating the most significant and impactful waste identified in the VSM, with a defined deadline for achieving the target [32,33].

##### Identified Issue and Improvement Goal

As shown in step 2 of Figure 2, the main issue arises from ward staff being too busy to answer calls, resulting in significant delays and inefficiencies. The goal was to reduce the number of phone calls taking more than 30 seconds to be answered by 50% within 3 days.

#### Step 4: Analyze the Root Cause

##### TPS Thinking 4

The root cause of operational problems is often attributable to waste within the process [32,33]. To identify this waste, TPS employs the “Five Whys” technique, which helps drill down to the root cause by repeatedly asking “why” [32,33].

##### Five Whys Analysis of Call Response Delays

As shown in Multimedia Appendix 3, the “Five Whys” method was applied to link the target with the VSM findings, enabling us to drill down into the key issue: “Why aren’t calls answered promptly?” Different formulations of the question may lead to different root causes.

After applying the “Five Whys,” it became clear that the root cause of the ward’s inability to answer phone calls was a shortage of staff. Additionally, because priority was given to direct patient care, the head nurse could not ensure that someone in the ward would be available to answer the phone immediately.

#### Step 5: Develop Countermeasures

##### TPS Thinking 5

Countermeasures should be developed based on the root cause and goals, prioritizing those that address major issues or are simple to implement. Ideally, the problem solver should take direct action on-site [32,33].

##### Proposed Countermeasures and Implementation

To address the root cause and achieve our goals, we developed 2 countermeasures. First, assign ward staff to regularly check and report bed availability at 4 scheduled intervals or proactively notify available beds. Second, if no staff are available, the bed control physician should personally check bed availability at the nursing station. Successful implementation of the first countermeasure reduces the time physicians spend making follow-up calls and meets the hospital superintendent’s expectations. During emergencies or when ward staff are unresponsive, the second countermeasure ensures immediate access to bed availability information. While implementing the first countermeasure, we simultaneously observed the situation and collected relevant numerical data.

#### Step 6: Implement Countermeasures and Observe at the Workplace

##### TPS Thinking 6

To verify results, it is necessary to return to the workplace where the problem originated, observe the implementation in practice, and collect numerical data [32,33].

Although the head nurse indicated that staff would be available at the nursing station to answer calls during the scheduled times, our implementation of the first countermeasure revealed a persistent issue identified in the VSM: frequent delays or failures of nursing and administrative staff in isolation wards to answer calls (Figure 2, step 2), particularly during evening shifts (data not shown). This forced bed control physicians to repeatedly call or contact other wards, creating a potential loop in steps 1 and 2. The delays were primarily due to reduced staffing during the evening shift (starting at 4:00 PM) and the absence of administrative personnel. Under these constraints, assigning staff specifically for bed availability checks was not feasible.

When the ward cannot answer the phone, the second countermeasure effectively locates available beds but has notable drawbacks. First, bed control physicians moving between nursing stations violates infection control protocols. Second, real-time updates on bed availability cannot be provided to administrators when changes occur. To address these issues, TPS principles were applied to optimize existing resources without increasing staff.

##### TPS Thinking 7

Prioritize process improvement and equipment optimization before considering an increase in workforce; adding staff should be the last resort [32,33].

To improve bed management efficiency, we conducted a detailed review of the VSM (Figure 2). We found that the primary method for checking bed availability involved ward staff searching for empty beds on the ward’s TV screen (step 3). This indicated that the hospital’s bed management system was already digitized, suggesting the presence of a database tracking bed occupancy and patient information.

Following this discovery, we consulted the Medical Affairs Office, which oversees admissions and discharges. They confirmed that the hospital information system (HIS) includes a bed inquiry platform containing essential inpatient data.

### The Second Cycle of TBP: Usability Testing of Existing Digital Platform

#### Step 1: Clarify the Problem

##### Expectations

Bed control physicians expect the hospital bed inquiry platform to provide all information required by the authorities.

##### Current Situation

The hospital bed inquiry platform displays a menu (see Multimedia Appendix 4) and returns 761 bed records upon accessing the system. Each record includes 28 fields, such as bed number, patient name, medical record number, gender, admission date, discharge date, and attending physician. Of the 761 beds, 399 are for acute care, while the remaining beds comprise deactivated beds (n=215), postoperative recovery beds (n=6), dialysis beds (n=36), emergency/virtual observation beds, long-term respiratory care beds, psychiatric day care beds, and nursing home beds.

When reviewing available beds using the platform’s built-in menu, 488 active beds are displayed, each with 28 fields. However, we only needed information for 148 beds, with just 3 relevant fields: bed number, medical record number (for occupancy), and gender. This demonstrates that the system provides unnecessary information.

The platform has a query function that allows bed availability checks by ward (see Multimedia Appendix 5). All combinations of the inquiry functions were tested. Currently, identifying double-occupancy rooms requires consulting a reference table. To find gender-specific beds, users must manually scan the 488 active beds, visually tracking the relevant data by moving the mouse pointer across the screen.

This inefficiency demonstrates that the current system does not meet physicians’ expectations, highlighting a significant issue that needs to be addressed.

##### Impact

Delays in determining bed availability jeopardize patient safety and the quality of care.

#### Step 2: Break Down the Problem

**Figure 3:**
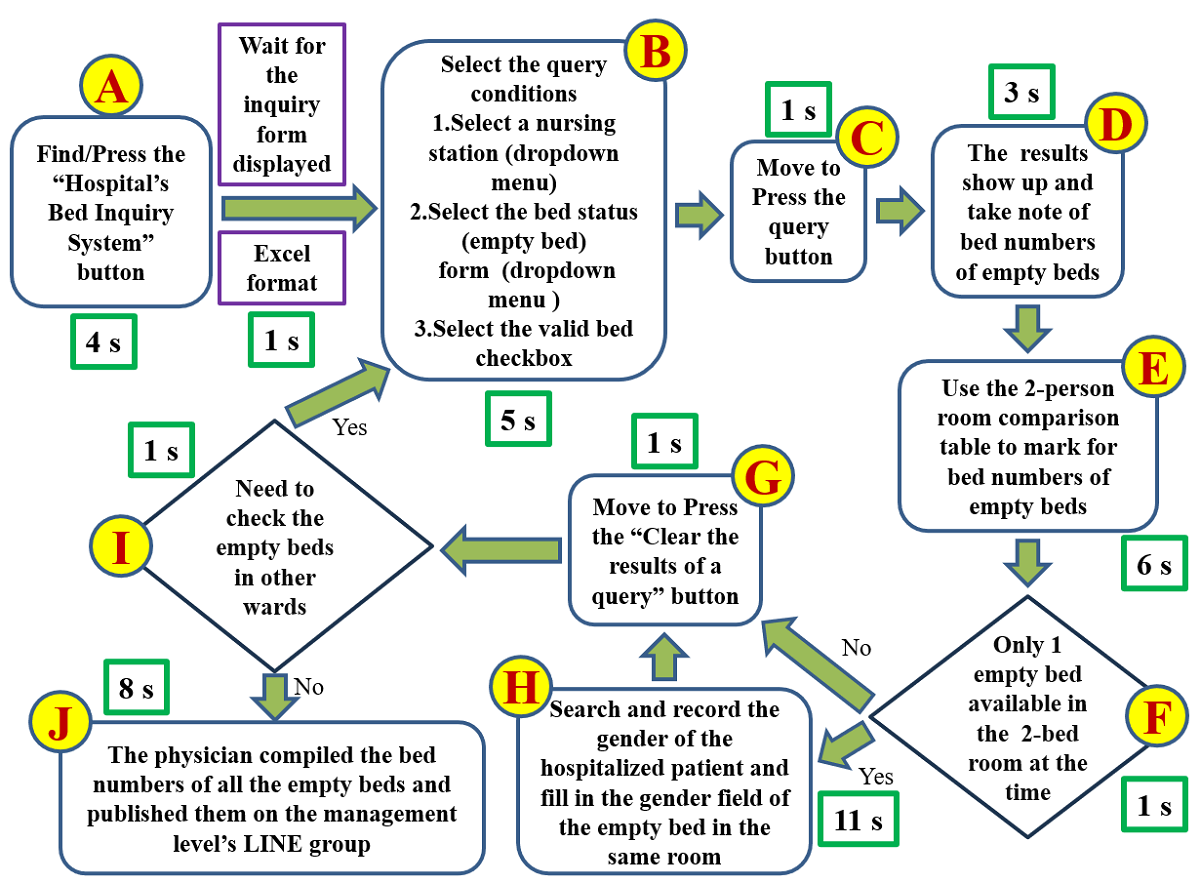
The value stream map illustrates the built-in query function of the hospital bed inquiry platform, which is used to check bed availability across the hospital. The times shown (in seconds [s]) represent the fastest possible query duration for a physician proficient in computer operations, assuming only 1 available bed is found. These values indicate the minimum time required to complete the query process.

Figure 3 illustrates the VSM, highlighting the most efficient method we identified for querying available beds hospital-wide. The times, measured in seconds, reflect the minimum duration required for a proficient physician to locate a single available bed. Steps E and H were the most time-consuming, taking 17 seconds to identify 1 bed. If multiple beds are available (eg, 10), the time required for steps E and H increases substantially.

#### Step 3: Establish a Target

Our goal is to reduce the time spent on steps E and H by 50% within 3 days. The rationale is as follows: As shown in Figure 3, the VSM revealed that steps E and H were the most time-consuming elements in the existing hospital bed inquiry platform. Together, these steps took 17 seconds for a single bed, and the time increased substantially when multiple beds were available.

The rationale for targeting a 50% reduction in these steps was straightforward: they represented the major bottlenecks and sources of waste—manual effort, visual scanning, and reliance on external tables—within an already digitized but inefficient bed inquiry process. The goal was to make the system more usable and efficient for physicians, moving closer to the “immediate and accurate information” expected by leadership.

A secondary subgoal was to reduce the total process time—from step A to step J—for a single bed in an isolation ward (42 seconds) by 50% within 6 days.

#### Step 4: Analyze the Root Cause

To identify double-occupancy rooms, we currently rely on a comparison table (Figure 3, step E). In collaboration with the Medical Affairs Office, we applied the “Five Whys” method to pinpoint the root cause (see Multimedia Appendix 6). The challenge arises because double-room designations depend on real-time factors, such as available equipment and ward conditions, preventing the establishment of a standardized guideline. Consequently, identifying double-occupancy rooms still requires referencing the double-room comparison table.

We applied the “Five Whys” method to determine the root cause of step H (data not shown). The root cause is that the hospital’s information system cannot automatically identify the gender of the patient in the adjacent bed in double rooms.

#### Step 5: Develop Countermeasures

Based on the root cause analysis and established targets, 2 countermeasures were proposed. First, for step E, bed control physicians were tasked with memorizing the comparison table to expedite data comparison and potentially reduce processing time. Second, for step H, Medical Affairs Office staff were instructed to input the patient’s gender into the bed management system during admission, resolving the gender mismatch issue in double-occupancy rooms. The second countermeasure involved enhancing physicians’ proficiency in quickly searching bed numbers and patient gender in the system, further reducing task completion time. During implementation, we continuously monitored the process and collected relevant data to evaluate the effectiveness of these measures.

#### Step 6: Implement Countermeasures and Observe at the Workplace

##### Challenges in Using the Current Bed Management Process

After the actual operation, bed control physicians found the process excessively complicated. Checking the availability of a single isolation nursing station required at least 10 steps, involving multiple mouse clicks and selections, leading to eye and wrist strain. The method still relied on manually using the comparison table to determine and record gender, missing opportunities to leverage the computer system’s advantages, such as speed, real-time processing, and automation. Even for experienced physicians, memorizing the double-room comparison table proved difficult. Consequently, no one opted to use these built-in functions for checking hospital-wide bed availability, highlighting the need for alternative solutions or improvements.

##### TPS Thinking 8

Problems arise in the workplace, and the solutions to these problems must also be found there [32].

### The Third Cycle of TBP: Solution Development

#### Step 1: Clarify the Problem With the Hospital Bed Inquiry Platform’s Built-In Features

##### Expectation

We expected to leverage the hospital’s computer information system to enable faster, repeatable, and more immediate bed availability inquiries, addressing staff shortages and the urgent need for quick bed assignments.

##### Current Situation

The hospital bed inquiry platform lacked real-time bed availability features and administrative access, limiting its functionality to meet our needs. However, upon further examination, we discovered an export function (Multimedia Appendix 4) that generates a Microsoft Excel (.xls) file with 761 entries across 28 columns, referred to as the “Exported Raw Data .xls,” as shown in Multimedia Appendix 7.

#### Step 2: Break Down the Problems in the Exported Raw Data .xls

**Figure 4:**
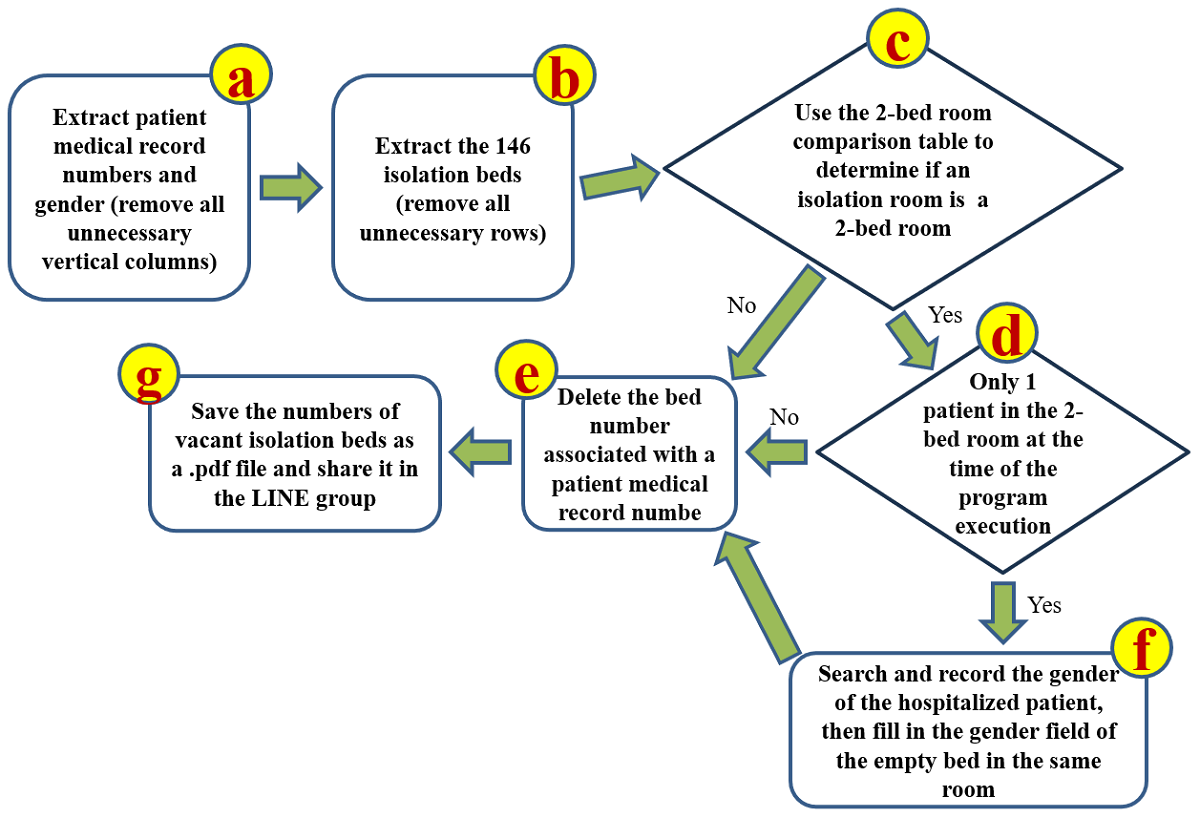
Value stream map for identifying vacant beds from the Exported Raw Data.xls file across the hospital. The strategy focuses on filtering out unnecessary information and retaining only the bed numbers of the 148 isolation beds, along with the patients’ medical record numbers and gender. For double-occupancy rooms, if only 1 patient is present at the time of execution, the gender of the admitted patient must be considered when displaying the vacant bed number.

Upon reviewing the exported data, we identified that most of the information was unnecessary. Of the 761 total entries, only 148 active bed numbers, provided by the Medical Affairs Office, were relevant. Similarly, of the 28 columns per entry, only 3 were pertinent: bed number, patient medical record number, and gender. The process for identifying vacant beds involved filtering the data down to these 148 entries and focusing on the 3 key columns. A bed was considered available if there was no associated medical record number. For 2-bed rooms, the gender of the admitted patient had to be considered, as only patients of the same gender can share a room. Figure 4 illustrates the VSM used to identify vacant beds from the exported raw data (.xls) across the hospital.

#### Step 3: Establish a Target

The goal is to develop a user-friendly Quick Isolation Bed Inquiry System within 3 days, leveraging data from the exported raw data (.xls) file. The system should allow anyone—not just physicians—to access real-time bed availability, enabling bed control physicians to quickly relay this information to hospital administrators.

#### Step 4: Analyze the Root Cause of Exported Raw Data .xls

The excess of irrelevant data in the exported file significantly hinders the efficient identification of available beds. Only the bed number, patient medical record number, and gender are considered essential and valuable for this process.

#### Steps 5 (Develop Countermeasures) and 6 (Implementing Countermeasures)

Based on the identified root cause and established targets, our countermeasure was clear: we needed to use the computer information system to eliminate unnecessary information. We explored 2 approaches: manual processing in Excel and automation using Visual Basic for Applications (VBA) to implement this plan through actionable steps.

The first countermeasure (manual processing in Microsoft Excel) involved the bed control physician manually handling the exported data file in Microsoft Excel. The steps were as follows:

- Delete the first 2 rows and unnecessary columns, retaining only the bed number, patient medical record number, and gender.
- Use the “Deactivated” column (cell N1) as a sorting header, then sort and remove all rows marked “Yes (Y),” leaving 488 usable beds.
- Filter these beds to retain only those from the A9, A8, A7, and A6 wards.
- Use the double-room comparison table to mark the gender of patients in double-occupancy rooms. If a room is partially occupied, the gender of the patient must be noted when displaying the vacant bed.
- Finally, sort by the medical record number column (cell I1) and remove all occupied beds. The remaining vacant beds are then recorded and shared in the LINE group.

**Figure 5:**
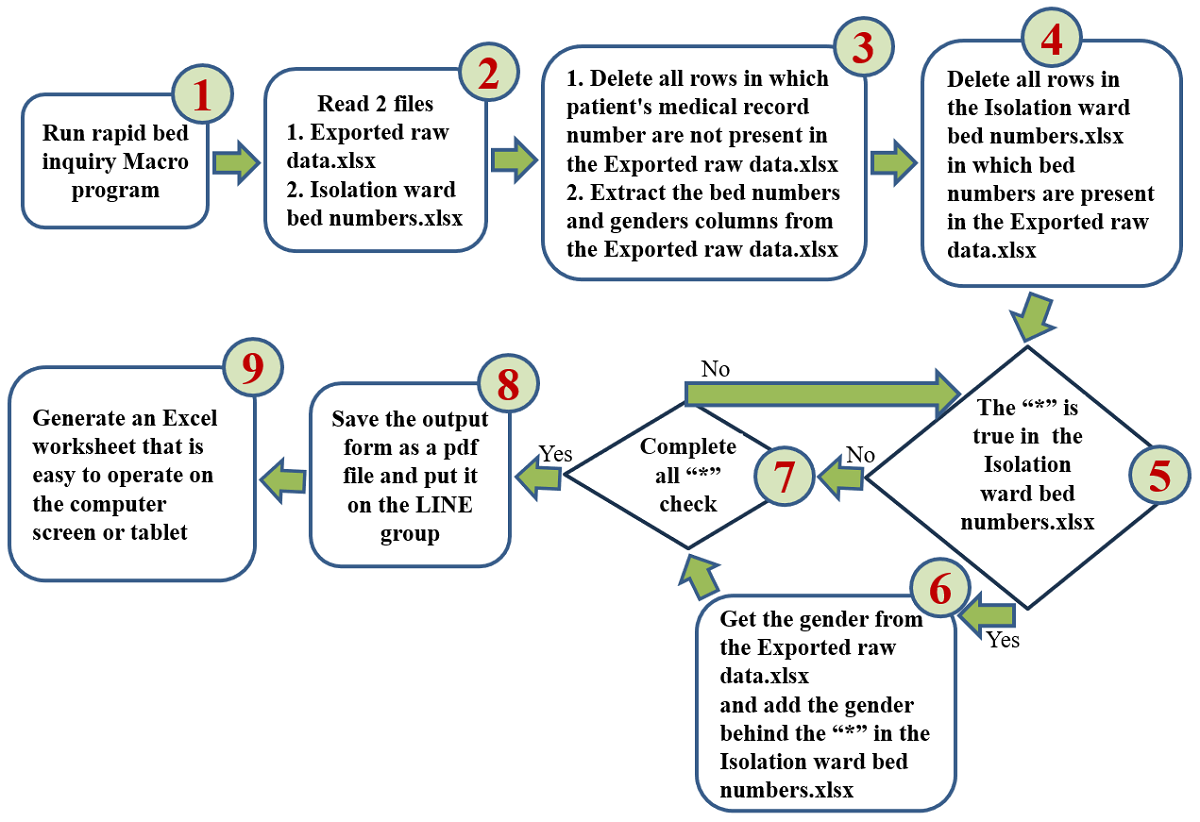
An algorithm based on value stream mapping techniques was used to develop a Visual Basic for Applications macro in Excel to automate the manual processes of sorting, deleting, and comparing data.

The second countermeasure (automated process using VBA) utilized Microsoft Excel’s VBA to automate the manual process. Drawing on prior experience developing a surgical scheduling system with VBA, we recognized the potential to streamline this task by creating a macro [30,44]. Before coding, we drafted an algorithm using the technique of constructing a VSM, converting the manual steps into a computer-executable workflow [44,45]. The algorithm in Figure 5 served as the blueprint for the VBA macro, automating the sorting, deletion, and comparison tasks of the first countermeasure.

**Figure 6:**
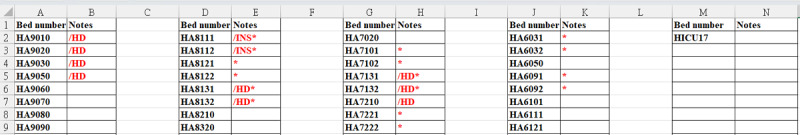
A simplified version of the Isolation Ward Bed Numbers.xlsx format. When the program runs, columns DE, GH, JK, and MN are merged into column AB. Column A lists the available bed numbers, while column B (the Notes column) records bed characteristics. For example, an asterisk (*) indicates a 2-bed room, “HD” denotes a dialysis-ready bed, and “INS” signifies that the patient in the adjacent bed has mental instability.

During program development, we anticipated fluctuations in the number of isolation ward beds due to changes in patient numbers or nursing staff availability. To manage this, we stored the current 148 bed numbers in a separate file, Isolation Ward Bed Numbers.xlsx. Any future updates to bed numbers would require changes only to this file. An adjacent “Notes” column was used to provide key details about each bed. Double-occupancy rooms were marked with an asterisk (*), while beds equipped for dialysis were labeled “HD” (hemodialysis), as illustrated in Figure 6.

For further reference, Multimedia Appendix 8 provides supplementary explanations of key concepts related to TPS thinking and the TBP methodology.

### Measurable Outcomes and Evaluation

The system was evaluated with a focus on process performance, consistent with quality improvement principles and the TPS, in which data collection is used to measure improvement rather than for accountability [32,33,42,43]. Both quantitative and qualitative metrics were applied to assess the process, identify waste, and compare improvements across the 3 cycles: the manual phone-call process (cycle 1), the HIS built-in platform (cycle 2), and the final automated Microsoft Excel VBA system (cycle 3).

Key quantitative metrics included the time required to complete a query (measured in seconds) and the total number of steps involved. These were supplemented by qualitative observations and VSM to identify inefficiencies, such as unanswered calls or excessive mouse clicks.

The final automated system was evaluated against prespecified primary and secondary outcomes to confirm its effectiveness. Primary indicators included process efficiency, system reliability (100% successful queries with no errors), and user accessibility (available 24/7). Process efficiency was evaluated using 2 criteria: (1) each cycle had to reduce the total number of steps by at least one compared with the previous cycle, and (2) the total process time had to be reduced by at least 50%. Secondary indicators included staff time savings and 100% accuracy in reporting bed status. Collectively, these metrics confirmed the system’s ability to provide a just-in-time, reliable, and user-friendly bed inquiry process.

### Ethical Considerations

The project underwent administrative review and received approval from hospital leadership in line with institutional standards for operational improvement initiatives. Under applicable national regulations and institutional guidelines, formal ethical approval was not required because the work constituted a quality improvement activity focused on evaluating internal procedures, without involving direct interventions on human participants [46,47]. The study was conducted in compliance with the Personal Data Protection Act [46], particularly Chapter I, Article 6, which permits the use of health-related data for medical and public health purposes, provided that individuals cannot be identified. Taiwan’s Constitutional Court, in a decision dated August 12, 2022, confirmed that Article 6, Paragraph 1 of the Personal Data Protection Act—which permits the use of health-related data for medical and public health purposes, provided that individuals cannot be identified—is constitutional [48]. The Court further found that the proviso in Article 6, Paragraph 1, Subparagraph 4 does not violate the principle of legal clarity or the principle of proportionality and is consistent with the protection of informational privacy under Article 22 of the Constitution [48]. In accordance with these regulations and the hospital’s internal requirements, informed consent was not required (Multimedia Appendix 9) [47]. To ensure privacy, all information used was anonymized, and no individual can be identified in the figures or appendices included in this manuscript. The system processes only bed numbers and gender from an internal HIS export; no patient-identifiable information is stored or transmitted outside the hospital’s secure network. No remuneration was provided, as the project did not involve the direct participation of human participants.

## Results

### Step 7: Evaluate the Results and the Process

#### Final System Functionality and Output Forms

Within 3 days of coding and verification, the Microsoft Excel macro was fully functional. Its interface was intentionally simplified to enhance accessibility, allowing users to press a single large button on the worksheet to automatically generate 3 distinct forms in approximately 4 seconds (discussed in the next section).

#### Final Interface

**Figure 7:**
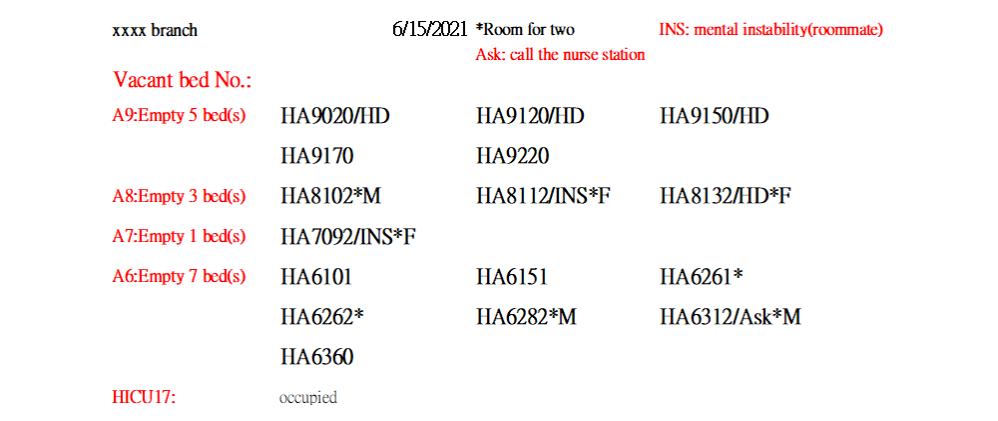
PDF form exported for upper management reference. An asterisk (*) indicates a 2-bed room, “HD” denotes a bed equipped for dialysis, and “INS” signifies that the patient in the adjacent bed has mental instability.

- PDF Form: Designed for upper management, this form is automatically exported in PDF and shared via the LINE group for quick reference (Figure 7).
- Large-Screen Form: Tailored for bed control physicians, this version is optimized for viewing on large screens such as computer monitors or tablets (Multimedia Appendix 10). The space below the bed numbers in the worksheet allows users to enter and store basic patient information and diagnoses for future reference.
- Smartphone Form: This mobile-friendly version is designed for bed control physicians to use on smartphones (Multimedia Appendix 11).

**Figure 8:**
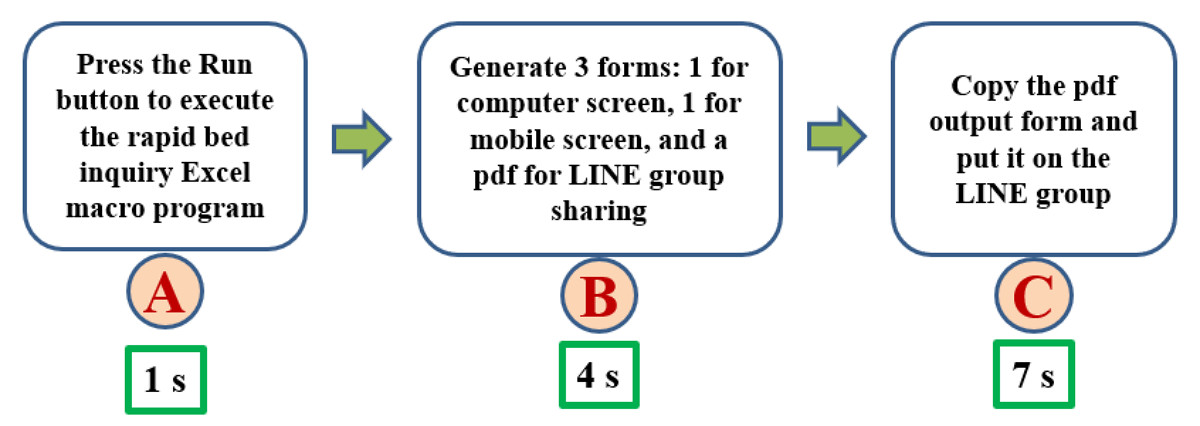
The completion of the isolation ward bed inquiry Excel macro has significantly streamlined the process for bed control physicians to quickly query available beds, resulting in an updated value stream map that reflects the improved workflow.

The completion of the isolation ward bed inquiry Microsoft Excel macro has greatly improved the efficiency of bed control physicians in querying available beds, resulting in an updated VSM that illustrates the enhanced workflow (Figure 8). After downloading the Exported Raw Data.xls, the macro generates 3 output forms in as little as 4 seconds (step B), and the fastest PDF report is sent to the LINE group in 7 seconds (step C). The macro also prevents duplicate file names by incorporating the execution date and time (down to the second) into each file name.

### Step 8: Standardize and Share the Successful Process

The Microsoft Excel macro generates a PDF and 2 bed availability forms with a single click, providing an efficient solution that meets the needs of bed control physicians while fulfilling hospital management’s expectations for quick and accurate information. The program was stored in a designated hospital system folder, allowing authorized personnel to run it remotely via a virtual private network at any time. It has also been adapted for Microsoft Office 365 and is publicly accessible (Multimedia Appendix 12).

### Process Performance Improvements and Achievement of Outcomes

The 3-cycle TBP process yielded a dramatic improvement in the speed, reliability, and usability of the bed inquiry system. The final automated solution reduced the process time by 97.4% (n=442 seconds saved/N=454 baseline seconds) compared with the original manual method. A comparative analysis of performance metrics across the 3 cycles is presented in Table 1. The final system successfully met all prespecified primary and secondary project outcomes, as detailed in Table 2.

**Table 1:** Comparative performance of the bed-inquiry process across 3 Toyota business practice cycles. This table summarizes outcomes from the value stream maps for the initial manual process (cycle 1; Figure 2; times represent best-case scenarios), the HIS^a^ built-in platform (cycle 2; Figure 3; tested by an experienced physician), and the automated Microsoft Excel VBA^b^ macro–based system (cycle 3; Figure 8).

Performance metric	Cycle 1: Manual phone-call process	Cycle 2: HIS built-in platform	Cycle 3: Automated Excel VBA system
Primary workflow	Sequential phone calls to 4 wards	Multistep navigation through the HIS graphical user interface	Single-click execution of a Microsoft Excel macro
Number of steps	25 steps for 4 wards	34 steps for 4 wards	3 steps after data export
Time to complete query	454 seconds (4 wards)	144 seconds (4 wards)	12 seconds (4 wards)
Time reduction from baseline	Baseline	68.3% (n=310 seconds saved/N=454 baseline seconds)	97.4% (n=442 seconds saved/N=454 baseline seconds)
Key inefficiencies/notes	Unanswered calls, manual data recording, and verbal communication	Excessive data, manual cross-referencing for double rooms, and multiple mouse clicks and menu selections	None identified; process is fully automated
System reliability and accuracy	Low; prone to human error, delays, and inconsistent information	High accuracy but dependent on correct manual navigation	100% (automated, repeatable process minimizes human error)
User accessibility	Limited; dependent on staff availability at specific times	High; accessible via the hospital network but with usability barriers	24/7; remotely accessible via a virtual private network

^a^HIS: hospital information system.

^b^VBA: Visual Basic for Applications.

**Table 2:** Achievement of prespecified project outcomes by the final automated system.

Outcome category and indicator			Target		Result		Status		
**Primary**									
	Process efficiency (steps)	Reduction of at least one step per cycle		Cycle 1 (25 steps) to cycle 3 (3 steps)		Achieved			
	Process efficiency (time)	>50% reduction		97.4% (n=442 seconds saved/N=454 baseline seconds) reduction		Achieved			
	System reliability	100% successful queries		100% (automated process)		Achieved			
	User accessibility	24/7 availability		24/7 via a virtual private network		Achieved			
**Secondary**									
	Staff time savings	Implied reduction in physician time		Significant reduction in manual query time		Achieved			
	System accuracy	100%		100% (data directly from HIS^a^ export)		Achieved			

^a^HIS: hospital information system.

### Improve Continuously

The Microsoft Excel macro can be adapted for various applications with minor modifications or additional functions. For example, incorporating ward codes allowed us to track availability and occupancy in each ward, and adding an attending physician’s list enabled us to monitor how many patients each physician was responsible for in different wards (see Multimedia Appendix 13). This demonstrates how small adjustments can extend the macro’s functionality, reflecting the principle of continuous improvement.

## Discussion

### Principal Findings

This study demonstrates that the structured TBP problem-solving process and TPS mindset facilitated the rapid creation of a Quick Isolation Bed Inquiry System, significantly improving bed allocation for patients with COVID-19. This user-centered system ensured timely admissions while meeting hospital requirements without the need for additional staff or expenses. The primary finding is the dramatic improvement in process efficiency. The final system reduced bed query time by 97.4% (from 454 seconds to 12 seconds) and streamlined the workflow from 25 manual steps to just 3 automated ones. A key insight emerged when the root cause of initial delays (staff shortages) could not be directly eliminated; the TPS mindset prompted a strategic pivot to leverage hidden value within the existing HIS, transforming a cumbersome data export function into an efficient, automated solution. This case provides a practical model of agile problem solving for resource-constrained health care environments. Opportunities for continuous improvement remain, including further automation through VBA integration and consolidation of manual steps, allowing for even greater time savings and resource optimization within the hospital’s infrastructure.

### Shared Problem-Solving Approaches in TPS and Health Care Systems

Outcomes are driven by actions, and actions arise from thinking. Although intangible, mindset shapes reasoning and direction, and is translated into tangible results through methods and tools [49-51].

### TPS Thinking and Health Care Parallels

TPS is rooted in systematic problem solving [31-36,49-52]. The first step is to “go to the source” and confirm problems on-site, based on the principle that all problems stem from waste [32,33]. By identifying and eliminating waste, efficient and low-cost solutions can be achieved [32-35]. Similarly, in health care, illness represents the problem, diagnosis confirms the issue, and treatment addresses the root cause [51,52]. Just as TPS links waste to root causes, physicians identify the underlying pathology before intervening. Treatments that resolve or control disease are “good actions,” paralleling TPS’s problem-solving mindset [48,51,52].

The structured diagnostic process mirrors TPS: history taking gathers subjective data, physical examination provides direct observations, and laboratory or imaging studies supply objective measures. These steps reflect TPS’s emphasis on observing the workplace and collecting accurate numerical data. Both approaches prioritize observation, root cause analysis, and targeted intervention [49-52].

### Transforming Health Care Through TPS Principles

The similarity in thinking explains why health care can readily adopt TPS tools [23-27,30,49,51,52]. Virginia Mason Medical Center demonstrated this by applying TPS to achieve transformative results [52]. Patient-centered care in hospitals mirrors TPS’s customer-focused philosophy, where success is defined by safe, effective, timely, affordable, and high-quality outcomes. Hospitals, therefore, must reduce costs, improve efficiency, and ensure quality, just as TPS strives for customer satisfaction in manufacturing [49,51,52].

Autonomation (automation with a human touch) ensures that machines or systems stop when abnormalities occur [27,32]. Hospitals apply this principle through medical devices that alarm when readings fall outside safety ranges and reporting systems that notify staff of abnormal test results. In our Isolation Ward Bed Numbers.xlsx program, debugging and error-flagging prevented mistakes and supported accurate bed allocation, embodying autonomation.

Just-in-time refers to delivering the right product at the right time [30,32]. In health care, this translates to timely medical services, exemplified by 24/7 emergency and surgical departments. Continuous staff availability ensures appropriate care whenever needed. These practices align with TPS values of respect for people, teamwork, and continuous improvement.

### Process Improvements by Eliminating Waste or Distilling Value

VSM is a lean tool for sequencing process steps, visualizing waste, and optimizing flow [32,33,36,49,51,52]. Three levels of VSMs were developed in this study, each highlighting waste in the bed inquiry process. The analysis revealed Toyota’s classical 7 wastes [30,32,33]: waiting, transportation, motion, overprocessing, inventory, overproduction, and defects. Table 3 summarizes each type of waste and its manifestation in the hospital bed inquiry workflow.

**Table 3:** Application of Toyota’s 7 wastes in the bed inquiry process.

Waste type	Definition in the Toyota production system	Example in the bed inquiry process
Waiting	Idle time when processes are delayed	Physicians waited up to 454 seconds for ward staff to answer phone calls before confirming bed status (Figure 2).
Transportation	Unnecessary movement of materials or information that is not essential to value-adding steps	Ward staff left phone stations to check monitors; physicians walked across wards to verify vacancies (Figure 2).
Motion	Excessive movement of people or equipment that does not add value to the product or service	Physicians performed repetitive clicks, navigated menus, and manually cross-referenced tables in the hospital’s inquiry system (Figure 3).
Overprocessing	Performing more work or steps than necessary, overly complex tasks, or exceeding customer requirements	The workflow involved 25 manual steps in cycle 1 (Figure 2) and 34 steps in cycle 2 (Figure 3), far beyond what was required.
Inventory	Excess stock, materials, or data beyond what is useful	The database listed 761 beds, but only 448 were clinically available for patient use, leading to confusion and inefficiency
Overproduction	Producing more data, information, or services than needed	The system displayed 28 fields and 761 records, although only 3 fields and 148 records were essential.
Defects	Any product or service that fails to meet specifications, resulting in rework, scrap, or correction	Manual transcription produced error-prone records, increasing the risk of inaccurate or incomplete bed information; inefficiencies persisted in cycles 1 and 2.

We were able to develop the Quick Isolation Bed Inquiry System only after extracting value from the VSMs in Figures 2 and 3 and creating a new VSM, as shown in Figure 8. This followed the unsuccessful attempts to eliminate waste in the first and second cycles. The final automated system systematically removed these wastes, resulting in a streamlined, efficient, and error-proof workflow (Figure 8). This step-by-step process of extracting value and eliminating waste provides a clear demonstration of problem decomposition through VSM and illustrates how processes can be improved to achieve all prespecified outcomes. Moreover, these iterative cycles highlight the importance of learning from unsuccessful attempts; without the inefficiencies revealed in cycles 1 and 2, the breakthrough in cycle 3 could not have been achieved. This underscores TPS’s core philosophy of continuous improvement, in which even failed experiments generate knowledge that guides the design of more effective and sustainable solutions.

### The TPS-Driven Approach as an Agile Paradigm for Health Care IT

This study demonstrated that a rapid, zero-cost solution can succeed where traditional health care IT projects often fail. Conventional IT implementations are frequently hindered by high costs, prolonged rollouts, and limited acceptance from clinical users [12,18,53-55]. By leveraging existing software (Microsoft Excel) and internal expertise, we avoided financial barriers and external vendors, while the direct involvement of bed control physicians in the design ensured seamless adoption, in contrast to the resistance often seen in top-down systems. The system was developed in only 3 days, contrasting with the lengthy timelines typical of commercial platforms. Notably, the 3 TBP cycles paralleled Agile software development, each serving as a short “sprint” with rapid prototyping, user feedback, and incremental improvement [55-59]. This iterative approach emphasized collaboration between users and problem solvers and illustrated the Agile principle of “responding to change over following a plan” [55-58]. These findings not only highlight the adaptability of TBP as a structured form of Agile in health care but also provide a practical model for resource-constrained settings, reinforcing the broader discussion on lean and user-centered innovation.

### Improve Continuously

After admissions of patients with COVID-19 ceased in August 2022, we easily adapted the Quick Isolation Bed Inquiry System’s Microsoft Excel macro for general ward use with minor modifications. This adaptation, detailed in Multimedia Appendices 13 and 14, has significantly enhanced bed allocation transparency and maximized utilization. Multimedia Appendices 13 and 14 demonstrate that the system can display key bed-related metrics—such as patient load per attending physician and per ward, total inpatients per ward and department, available beds per ward, and next-day discharge information (patients per ward and per attending physician)—based on input files. Crucially, the macro’s core program requires no modification for expansion. Simply inputting a file with physician names and their departmental affiliations (eg, surgical, internal medicine, or even the entire hospital) will display all relevant bed information for that specific group. This demonstrates the system’s inherent scalability beyond infectious disease scenarios.

### Impact on the Hospital’s Bottom Line

#### Overview

While we did not quantify the direct financial “bottom line” impact in monetary terms, the implications for health care are significant.

#### Improved Patient Flow

Faster bed allocation allows patients requiring admission (especially severe COVID-19 cases) to be moved into appropriate care settings more quickly. This reduces wait times in emergency departments or holding areas, which can indirectly lead to better patient outcomes and shorter lengths of stay.

#### Optimized Resource Utilization

Efficient bed management ensures that beds are not left vacant unnecessarily, maximizing the hospital’s capacity for patient care.

#### Reduced Staff Burnout and Increased Productivity

By reducing the time and mental strain staff spend on tedious bed inquiries, they can dedicate more time to direct patient care, consultations, and other critical duties. This boosts staff morale and overall productivity, helping to mitigate the impact of existing staff shortages. Although not a direct monetary saving, avoiding additional workforce costs was a key objective.

### Implications for Health Care Management and Future Directions

Our results point to valuable directions for practical health care delivery and policy development. This study provides a compelling argument for empowering frontline innovators, demonstrating that clinicians and staff can develop highly effective solutions when equipped with appropriate methodological tools. This fosters a culture of bottom-up innovation, which can be more agile and responsive than traditional top-down management approaches [54,56,60]. For hospital IT departments, this case serves as a call to re-evaluate existing IT assets for “hidden value.” Rather than focusing exclusively on procuring new systems, IT leaders should encourage periodic audits of existing platforms to identify underutilized functionalities—such as data export features—that can be leveraged for rapid, low-cost solution development. This also suggests a need for health care organizations to cultivate a “lean IT” culture that extends beyond clinical workflows into IT development and administrative problem solving. Such a culture would foster continuous improvement and provide staff with the training and autonomy to eliminate waste and distill value in digital processes.

To align with our commitment to continuous improvement and long-term system utility, we plan to conduct a comprehensive outcome evaluation of this innovation. Future efforts will focus on quantifying its impact on key performance indicators, including bed turnover rates, patient flow efficiency (eg, reduced wait times), and staff time savings. We will also administer user satisfaction surveys to gather both qualitative and quantitative feedback from bed control physicians, nursing staff, and administrators on usability, reliability, and workflow integration. In addition, we will evaluate the system’s long-term sustainability, including performance and maintenance requirements. Together, these outcome measures will provide robust evidence of the system’s effectiveness and enduring value, strengthening the case for impactful technology-enabled innovations in health care. Several avenues for advancement emerge from this work. Integrating the system with HIS through application programming interfaces, rather than relying on file exports, would enable true real-time updates. Incorporating machine learning algorithms could allow the prediction of bed availability based on historical patterns and current patient flow, shifting management from reactive to proactive. Furthermore, natural language processing could support voice-activated queries, further reducing user burden and enhancing accessibility.

### Limitations

#### Information Security and System Sustainability

This paper has 5 limitations. First, the use of VBA macros introduces potential information-security risks, a well-recognized concern in IT governance. However, these risks were proactively mitigated within a robust security framework: the system operates exclusively on the hospital’s secure intranet and is accessible only to authorized personnel via a virtual private network. Critically, the macro does not process, transmit, or store any patient-identifiable data outside institutional systems. These controls—together with standard institutional safeguards such as regular system patching and anti-malware protection—effectively mitigate the residual risks associated with macro-enabled files. This reflects a deliberate balance between urgent operational needs and security, offering a pragmatic model for rapid, frontline innovation within a controlled environment. Second, the long-term sustainability and compatibility of a VBA-based tool present a valid governance challenge, particularly amid ongoing Microsoft Office updates. We addressed this by framing the system’s lifecycle in 2 stages.

#### Stage 1: Proven Prototype (Current State)

The current tool functioned as a robust and effective prototype, with its durability demonstrated by a straightforward migration from Microsoft Excel 2016 to Microsoft 365 that required only minor modifications. By providing the updated and fully functional code in Multimedia Appendix 12, we confirm its ongoing usability.

#### Stage 2: Blueprint for Enterprise Integration (Future State)

Having demonstrated substantial value and impact, the tool now serves as both a proof-of-concept and a clear blueprint for our IT department to develop a permanent, integrated solution within the hospital’s enterprise infrastructure. This 2-stage approach addresses the long-term challenge by strategically transitioning the innovation from a rapid, tactical solution into a formal, sustainable system.

Third, as a single-center case study, our implementation was shaped by local institutional factors, such as an existing HIS export function and in-house familiarity with VBA. However, these factors do not limit the generalizability of the underlying methodology. We argue that the core contribution lies not in the specific tool (VBA), but in the agile, resource-conscious framework. The fundamental prerequisite for our approach is an information system capable of exporting data to a standard spreadsheet format (eg, .xlsx, .csv)—a near-universal feature of modern hospital systems. The choice of VBA was one of convenience during an emergency, leveraging a built-in, zero-cost tool. The system’s logic can be readily implemented in any modern programming environment capable of reading a spreadsheet file, including Python, R, C#, or Java. Thus, the approach is technology-agnostic and can be adapted to the existing technical expertise of any health care institution. The required skill is not “VBA expertise” but simply the ability to parse a data file—a straightforward task for any IT professional. This underscores that our problem-solving method is both highly accessible and transferable to a wide range of organizations facing similar challenges.

Fourth, while we demonstrated substantial improvements in process efficiency, this study did not measure the downstream impact on clinical or financial outcomes. We did not quantify changes in patient wait times, bed turnover rates, or overall length of stay, which represent important areas for future research.

Fifth, because most of the authors were also the system developers, a potential risk of evaluation bias exists. However, we contend that this risk was substantially mitigated by our study design and methodological approach. Our primary safeguard was the use of prespecified, objective, and quantitative metrics—specifically, query time and the number of process steps. These hard measures (eg, a 97.4% reduction, from 454 seconds to 12 seconds, in query time and a reduction from 25 steps to 3) are not susceptible to subjective interpretation. Furthermore, the dual implementer-evaluator role was a deliberate choice, aligning with established frameworks for rapid, real-world quality improvement, where this practice is common and often necessary. Our study’s design is consistent with the SQUIRE 2.0 guidelines for reporting quality improvement [39], which accommodate projects in which implementers evaluate their own work. The iterative process also mirrors the plan-do-study-act (PDCA) cycle [41] and Agile methodology [55], both of which rely on tight feedback loops in which the development team is intrinsically involved in evaluation to facilitate agile problem solving. While independent validation could further strengthen the findings, our methodology represents a well-supported approach in quality improvement science, ensuring the credibility of our results in this urgent, real-world context.

### Conclusions

By systematically applying the TPS mindset and the TBP framework, this study developed a zero-cost, user-centered Quick Isolation Bed Inquiry System. The intervention proved highly effective in a real-world hospital setting during the COVID-19 pandemic. All prespecified outcomes were achieved without incurring additional costs or requiring new staff, ensuring the timely admission of critically ill patients. This work offers a practical model for agile, bottom-up innovation in health care IT, demonstrating that leveraging underutilized functions within existing HIS can deliver rapid and impactful solutions, particularly in resource-constrained or crisis environments. Our findings contribute to the literature by providing a real-world example of applying TPS principles to overcome the high costs and lengthy implementation timelines typical of conventional health care IT projects. The system’s adaptability and scalability for broader hospital use have been confirmed postpandemic. Future research should quantify downstream impacts on clinical outcomes, such as patient wait times and bed turnover rates, to further validate the value of this approach.

## Appendix

Multimedia Appendix 1 Calculate the time spent (in seconds) querying the availability of vacant beds in wards A9, A8, A7, and A6, based on the value stream map shown in Figure 2.

Multimedia Appendix 2 Methodology and rationale for using "best-case" or "minimum-time" scenarios.

Multimedia Appendix 3 The "Five Whys" technique was used to identify the root cause of the problem: "Why are calls not answered promptly?".

Multimedia Appendix 4 This is the menu screen of the hospital bed inquiry platform. Pressing the "Query" button displays 761 bed records, each containing 28 fields.

Multimedia Appendix 5 The hospital bed inquiry platform's built-in query function allows users to check bed availability across different hospital wards.

Multimedia Appendix 6 In collaboration with the Medical Affairs Office, we applied the "Five Whys" method to identify the root cause of the problem: "Why must we use the double-room comparison table to identify double-occupancy rooms?".

Multimedia Appendix 7 A simplified version of the Exported Raw Data.xls file. Of the 28 available columns, only 3—highlighted in red—are required for use.

Multimedia Appendix 8 The following section offers supplementary explanations of key concepts related to Toyota production system (TPS) thinking and the Toyota business practice methodology. It serves as background material for readers who may be less familiar with TPS.

Multimedia Appendix 9 Scope of human research cases exempt from ethics committee review.

Multimedia Appendix 10 This large-screen form is designed for use by bed-control physicians on computers or tablets. An asterisk (*) indicates a 2-bed room, “HD” denotes a bed equipped for dialysis, and “INS” signifies that the patient in the adjacent bed has mental instability.

Multimedia Appendix 11 This smartphone form is designed for mobile use by bed-control physicians. An asterisk (*) indicates a 2-bed room, “HD” denotes a bed equipped for dialysis, and “INS” signifies that the patient in the adjacent bed has mental instability.

Multimedia Appendix 12 The original Excel macro code for the Quick Isolation Bed Inquiry System has been adapted for compatibility with Microsoft Office 365.

Multimedia Appendix 13 With minor adjustments, the Excel macro provides versatility for various applications. For example, integrating ward codes allows efficient monitoring of bed availability and occupancy across different wards, while adding an attending physician list enables tracking of patient loads for each physician. In the Notes column, an asterisk (*) indicates a 2-bed room, “HD” denotes a dialysis-equipped bed, and “INS” signifies that the patient in the adjacent bed has mental instability.

Multimedia Appendix 14 The Quick Isolation Bed Inquiry System’s Excel macro can be easily adapted for a variety of applications with minor modifications or additional functions. By incorporating ward or department codes, we were able to track bed availability and occupancy in specific areas. Adding an attending physician list also allowed us to monitor the number of patients for whom each physician was responsible across different wards. Additionally, the system can generate statistics on the number of patients in each ward expected to be discharged the following day.

## Abbreviations

HIS: hospital information system
PDCA: plan-do-check-act
SQUIRE: Standards for Quality Improvement Reporting Excellence
TBP: Toyota business practice
TPS: Toyota production system
VBA: Visual Basic for Applications
VSM: value stream map

## Data Availability

The data sets analyzed in this study are available from the corresponding author upon reasonable request.
