# Supplementary material for: Developing a Quick Isolation Bed Inquiry System During the COVID-19 Outbreak: User-Centered Design Approach Based on the Toyota Production System
Source: JMIR Form Res. 2025 Oct 17;9:e67152. doi: 10.2196/67152 (PMC12579300; doi:10.2196/67152)
Supplement: Multimedia Appendix 1 [file formative_v9i1e67152_app1.pdf]

Supplementary Table 1. Calculate the time spent (measured in seconds) on querying the availability of vacant beds in ward A9 based on the value stream map in Figure 2.

Time spent / Date	Time spent that the surgeon called the A9 ward, and a ward staff answered the phone				Time spent that the ward staff searched for all the empty beds on the TV screen and the surgeon wrote down the bed numbers of the vacant bed				Total amount of time spent
Time of obtaining data	8 AM	12 PM	4 PM	8 PM	8 AM	12 PM	4 PM	8 PM	
Day 1	6	8	7	9	21	16	19	15	101
Day 2	5	7	6	7	22	21	20	22	110
Day 3	5	6	8	6	13	10	8	10	66
Day 4	5	7	6	8	18	20	23	20	107
Day 5	6	7	7	9	25	22	19	22	117
Day 6	5	6	6	8	20	25	20	22	112
Day 7	5	6	6	7	20	25	28	23	120
**Mean**	5	7	7	8	20	20	20	19	105

Supplementary Table 2. Calculate the time spent (measured in seconds) on querying the availability of vacant beds in ward A8 based on the value stream map in Figure 2.

Time spent / Date	Time spent that the surgeon called the A8 ward, and a ward staff answered the phone				Time spent that the ward staff searched for all the empty beds on the TV screen and the surgeon wrote down the bed numbers of the vacant bed				Total amount of time spent
Time of obtaining data	8 AM	12 PM	4 PM	8 PM	8 AM	12 PM	4 PM	8 PM	
Day 1	7	8	7	8	27	25	20	18	120
Day 2	6	7	7	9	24	20	23	22	118
Day 3	6	8	8	9	21	19	24	20	115
Day 4	5	9	8	12	16	20	26	18	114
Day 5	6	7	7	8	23	26	28	23	128
Day 6	6	8	8	8	26	28	23	20	127
Day 7	5	8	6	9	21	23	26	23	121
**Mean**	6	8	7	9	23	23	24	21	120

Supplementary Table 3. Calculate the time spent (measured in seconds) on querying the availability of vacant beds in ward A7 based on the value stream map in Figure 2.

Time spent / Date	Time spent that the surgeon called the A7 ward, and a ward staff answered the phone				Time spent that the ward staff searched for all the empty beds on the TV screen and the surgeon wrote down the bed numbers of the vacant bed				Total amount of time spent
Time of obtaining data	8 AM	12 PM	4 PM	8 PM	8 AM	12 PM	4 PM	8 PM	
Day 1	10	8	7	8	26	18	16	21	114
Day 2	7	7	9	9	20	20	19	28	119
Day 3	8	8	7	8	18	21	16	20	106
Day 4	7	6	7	6	20	19	23	26	114
Day 5	7	7	7	9	19	22	21	20	112
Day 6	6	7	8	10	24	20	26	24	125
Day 7	7	8	7	8	24	25	27	30	136
**Mean**	7	7	7	8	22	21	21	24	118

Supplementary Table 4. Calculate the time spent (measured in seconds) on querying the availability of vacant beds in ward A6 based on the value stream map in Figure 2.

Time spent / Date	Time spent that the surgeon called the A6 ward, and a ward staff answered the phone				Time spent that the ward staff searched for all the empty beds on the TV screen and the surgeon wrote down the bed numbers of the vacant bed				Total amount of time spent
Time of obtaining data	8 AM	12 PM	4 PM	8 PM	8 AM	12 PM	4 PM	8 PM	
Day 1	8	8	7	7	19	22	21	20	112
Day 2	8	7	7	8	21	25	22	18	116
Day 3	6	7	9	6	20	16	19	20	103
Day 4	7	8	8	6	16	19	22	20	106
Day 5	6	6	7	8	22	21	18	18	106
Day 6	8	6	7	8	20	25	20	22	116
Day 7	7	7	7	8	21	23	25	20	118
**Mean**	7	7	7	7	20	22	21	20	111
