# Supplementary material for: Developing a Quick Isolation Bed Inquiry System During the COVID-19 Outbreak: User-Centered Design Approach Based on the Toyota Production System
Source: JMIR Form Res. 2025 Oct 17;9:e67152. doi: 10.2196/67152 (PMC12579300; doi:10.2196/67152)
Supplement: Multimedia Appendix 2 [file formative_v9i1e67152_app2.pdf]

This section clarifies our methodology and the rationale behind using "best-case" or "shortest time" scenarios.

Justify Best-Case Time Estimates

In line with TPS principles, we deliberately focused on "best-case" or "shortest time" process estimates throughout our analysis. This approach aimed to isolate and evaluate the irreducible minimum time required for value-adding steps, rather than quantify the full extent of inefficiencies caused by non-value-added activities such as unanswered calls or long wait times. These wasteful elements, although prevalent, were intentionally excluded as they represent conditions that should be eliminated in an optimized system.

For example, in TBP Cycle 1, Step 2, we measured the average of the shortest successful query attempts to highlight the inherent delay in even the most efficient manual processes. Similarly, in TBP Cycle 2, Step 2, we assessed the minimum time needed for a proficient user to identify a bed using the existing digital system, thereby exposing design-related inefficiencies under ideal conditions. This method allowed us to demonstrate that even in optimal scenarios, the existing workflows failed to meet urgent operational needs.

By identifying inefficiencies within the "value-added" steps themselves, this best-case analysis provided clear justification for system redesign and automation, supporting our overarching goal of lean improvement and waste elimination.

Address Unanswered Calls and Process Inefficiencies

A critical inefficiency in the original manual bed inquiry process was the high number of unanswered phone calls, particularly during evening shifts when staffing was limited. Although we did not quantitatively record the frequency or cumulative time lost due to unanswered calls, direct observation revealed that bed control physicians frequently had to redial or call multiple wards, as illustrated in **Figure 2**. These repeated attempts introduced significant delays and created feedback loops in the inquiry workflow.

In line with the TPS philosophy, such delays were classified as non-value-added activities, or "pure waste," which do not contribute to patient care or process effectiveness. Rather than measuring this inefficiency, our approach focused on its complete elimination. The newly developed automated system bypassed the need for phone calls entirely, addressing the root cause of these delays.

To assess the manual system's best-case performance, we reported the "average shortest query time," measured only when a call was successfully answered. Even in this optimal scenario, the process took an average of 454 seconds. This further justified the need for an automated, real-time system to improve bed inquiry efficiency and reliability.
