# Supplementary figures and images for: Developing a Quick Isolation Bed Inquiry System During the COVID-19 Outbreak: User-Centered Design Approach Based on the Toyota Production System
Source: JMIR Form Res. 2025 Oct 17;9:e67152. doi: 10.2196/67152 (PMC12579300; doi:10.2196/67152)

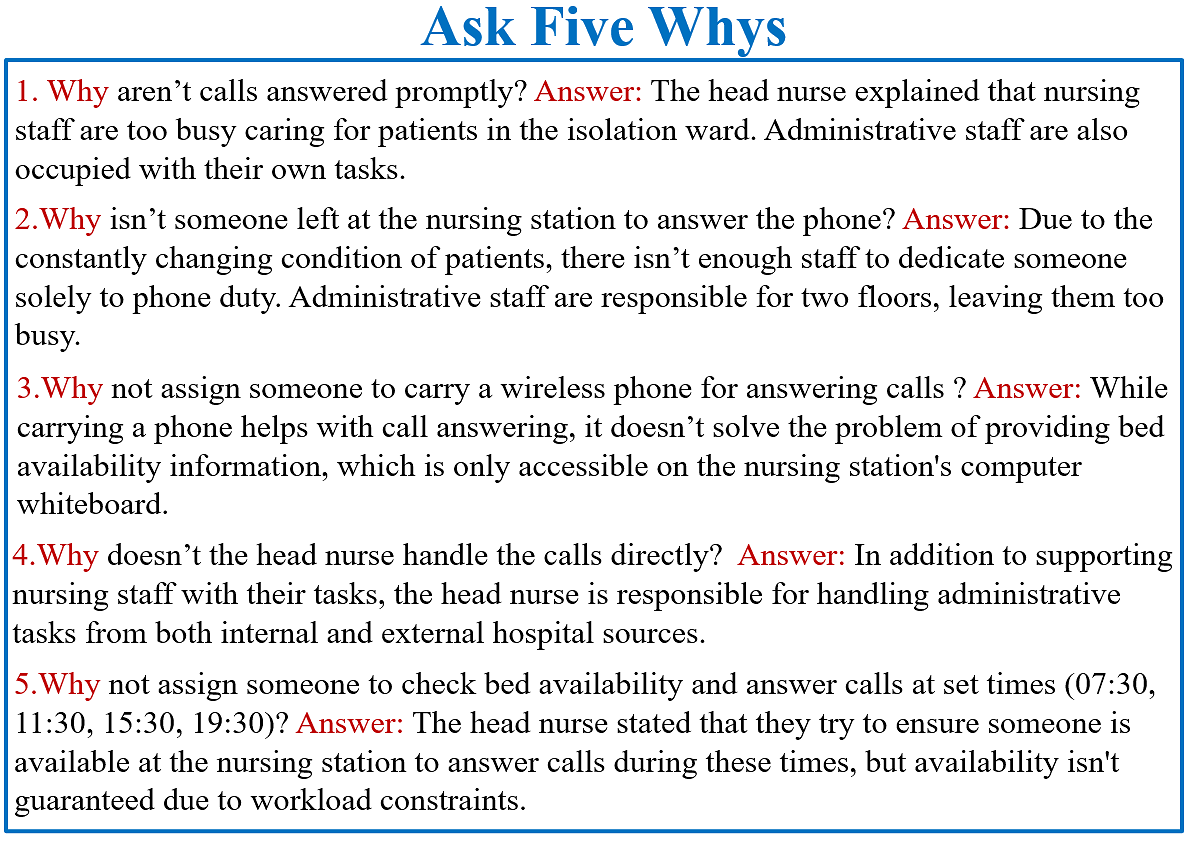

Supplement: Multimedia Appendix 3 [file formative_v9i1e67152_app3.png]

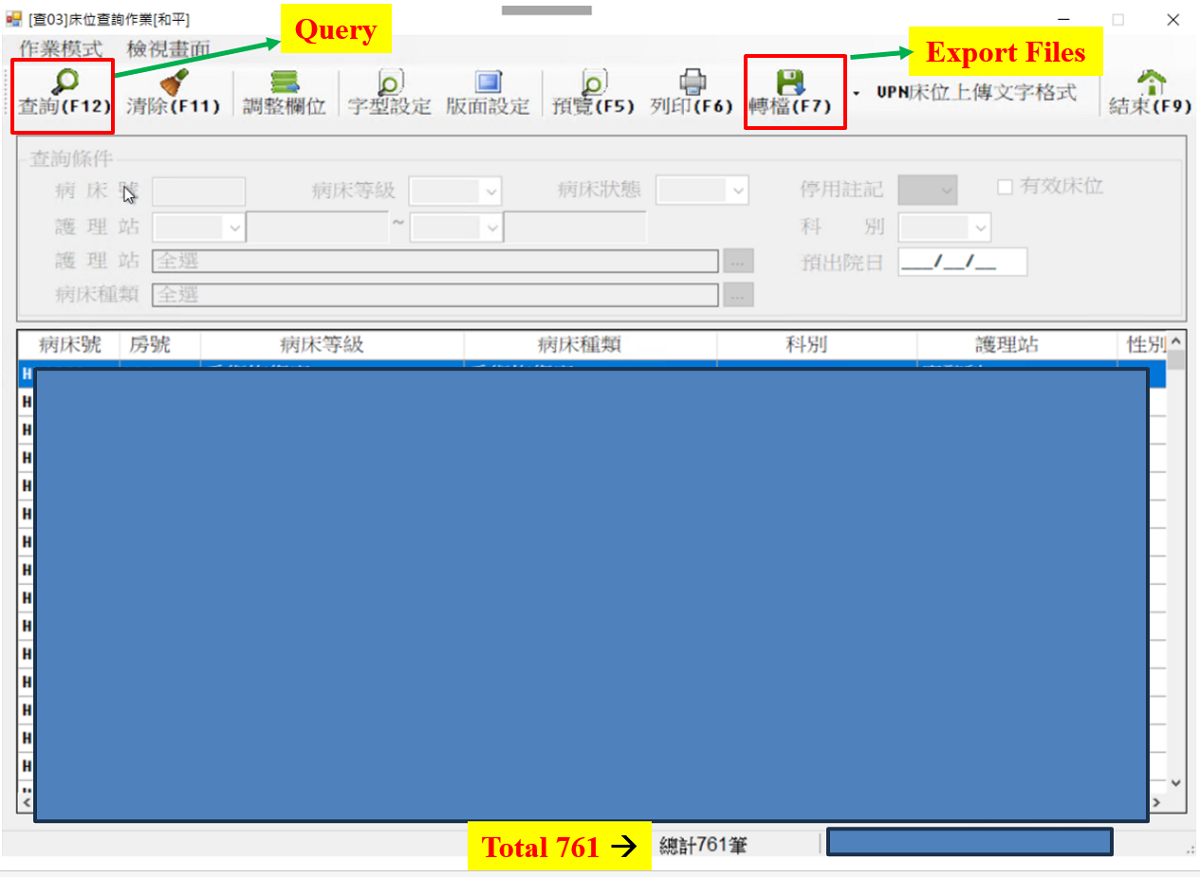

Supplement: Multimedia Appendix 4 [file formative_v9i1e67152_app4.png]

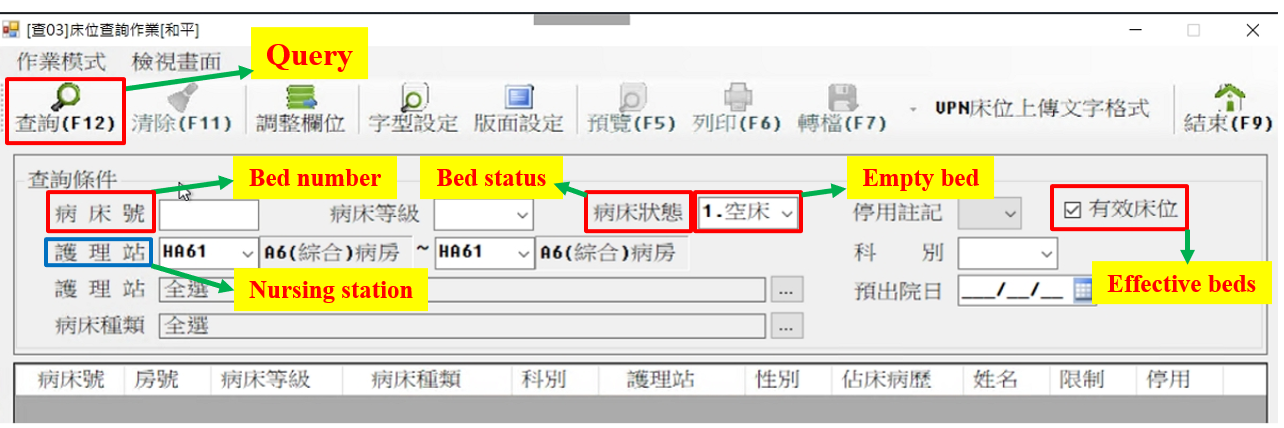

Supplement: Multimedia Appendix 5 [file formative_v9i1e67152_app5.png]

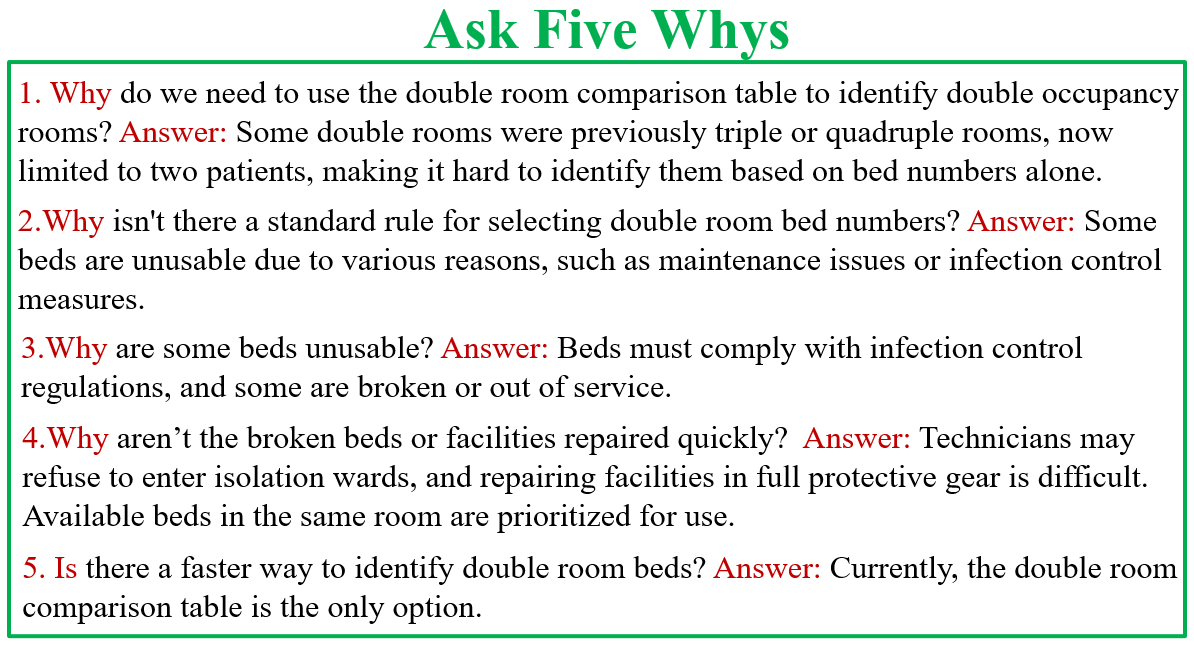

Supplement: Multimedia Appendix 6 [file formative_v9i1e67152_app6.png]

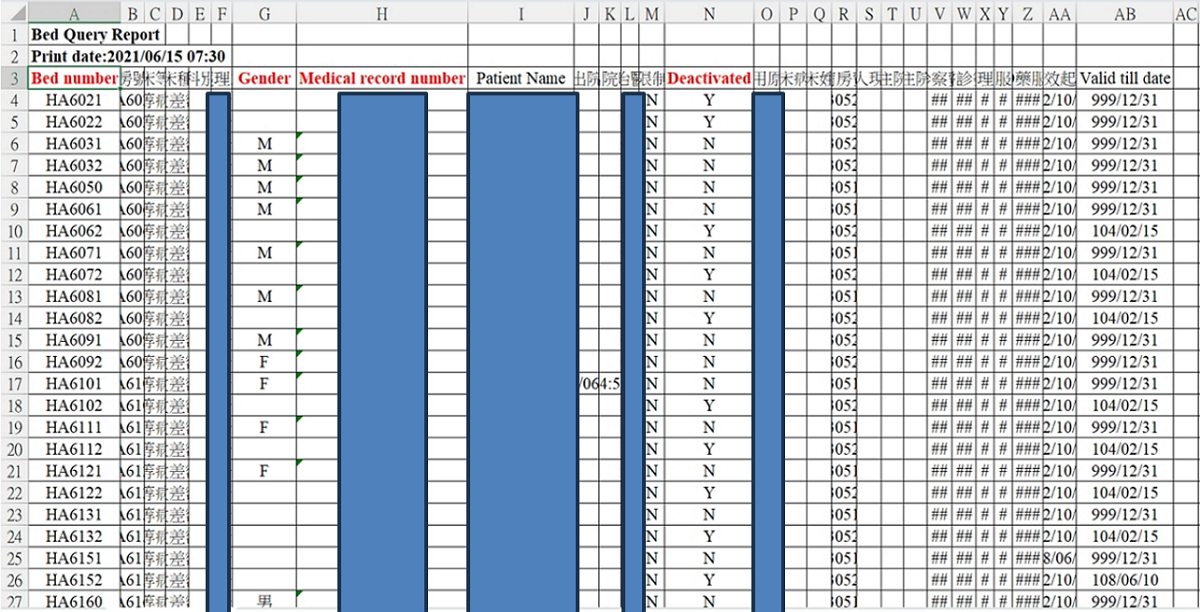

Supplement: Multimedia Appendix 7 [file formative_v9i1e67152_app7.png]

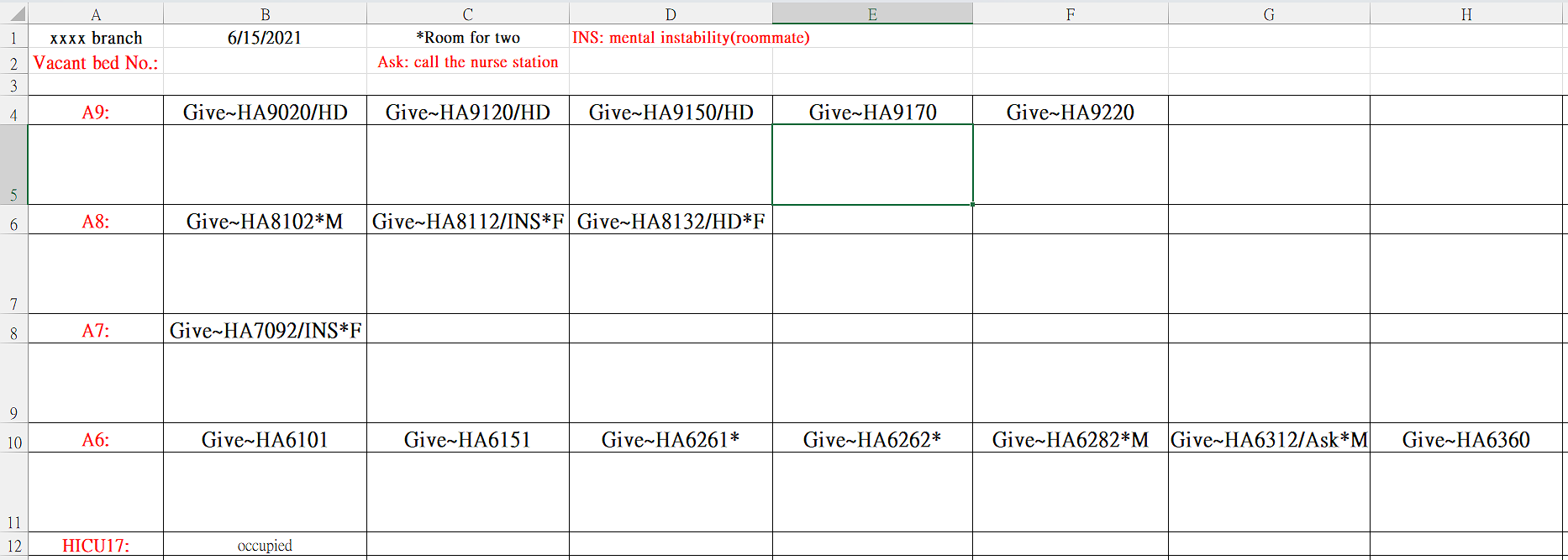

Supplement: Multimedia Appendix 10 [file formative_v9i1e67152_app10.png]

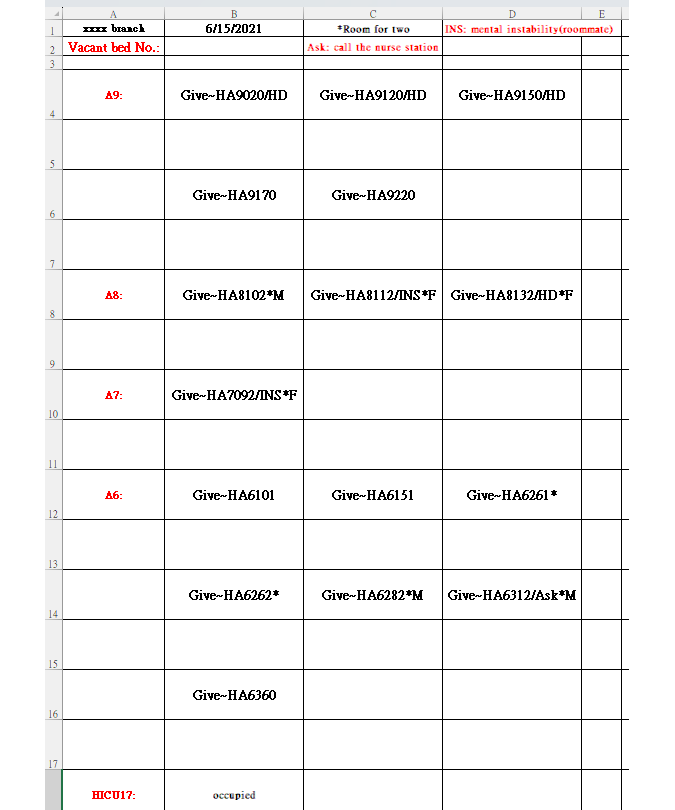

Supplement: Multimedia Appendix 11 [file formative_v9i1e67152_app11.png]

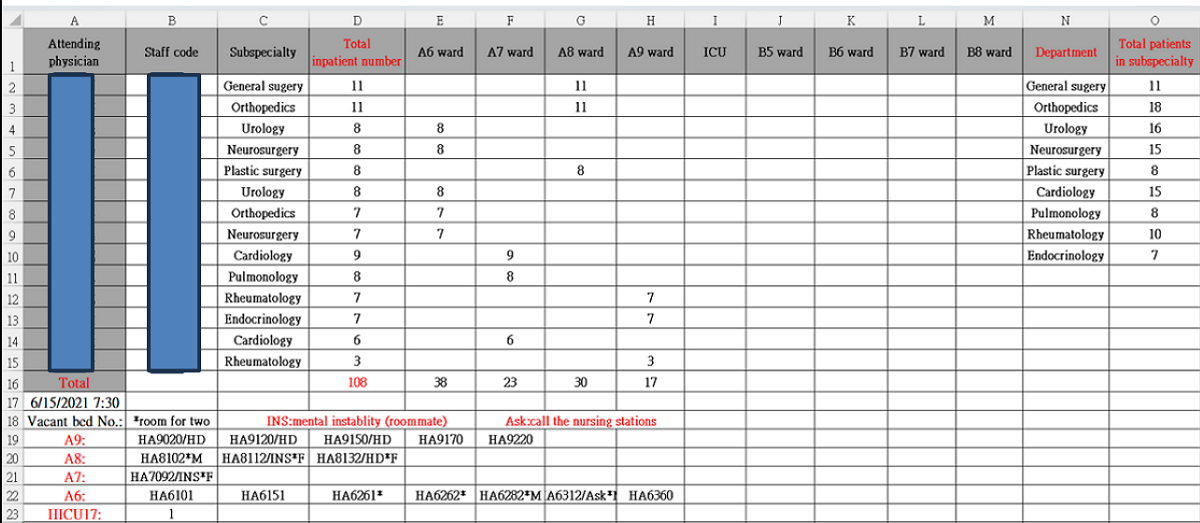

Supplement: Multimedia Appendix 13 [file formative_v9i1e67152_app13.png]

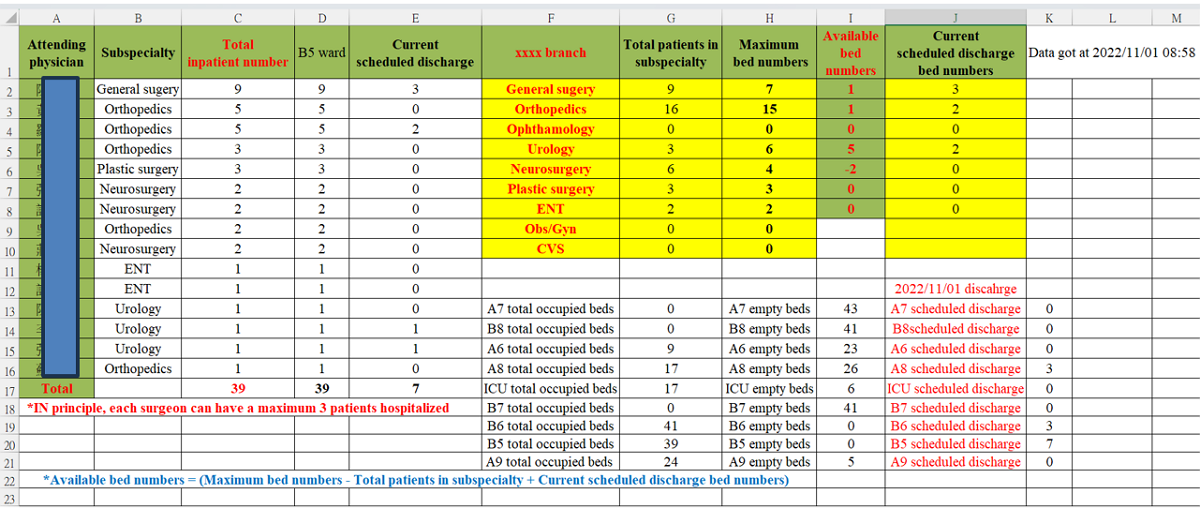

Supplement: Multimedia Appendix 14 [file formative_v9i1e67152_app14.png]
