# Supplementary material for: Developing a Quick Isolation Bed Inquiry System During the COVID-19 Outbreak: User-Centered Design Approach Based on the Toyota Production System
Source: JMIR Form Res. 2025 Oct 17;9:e67152. doi: 10.2196/67152 (PMC12579300; doi:10.2196/67152)
Supplement: Multimedia Appendix 8 [file formative_v9i1e67152_app8.pdf]

The following section provides supplementary explanations of essential concepts related to Toyota Production System (TPS) thinking and the Toyota Business Practice (TBP) methodology. It is intended as background material for readers who may be less familiar with TPS.

Highlight the key concepts of the TBP methodology

Figure 1 outlines the TBP problem-solving process, emphasizing the essential elements at each stage.

Figure 1. Overview of the Toyota Business Practice problem-solving process, detailing the critical components of each step.

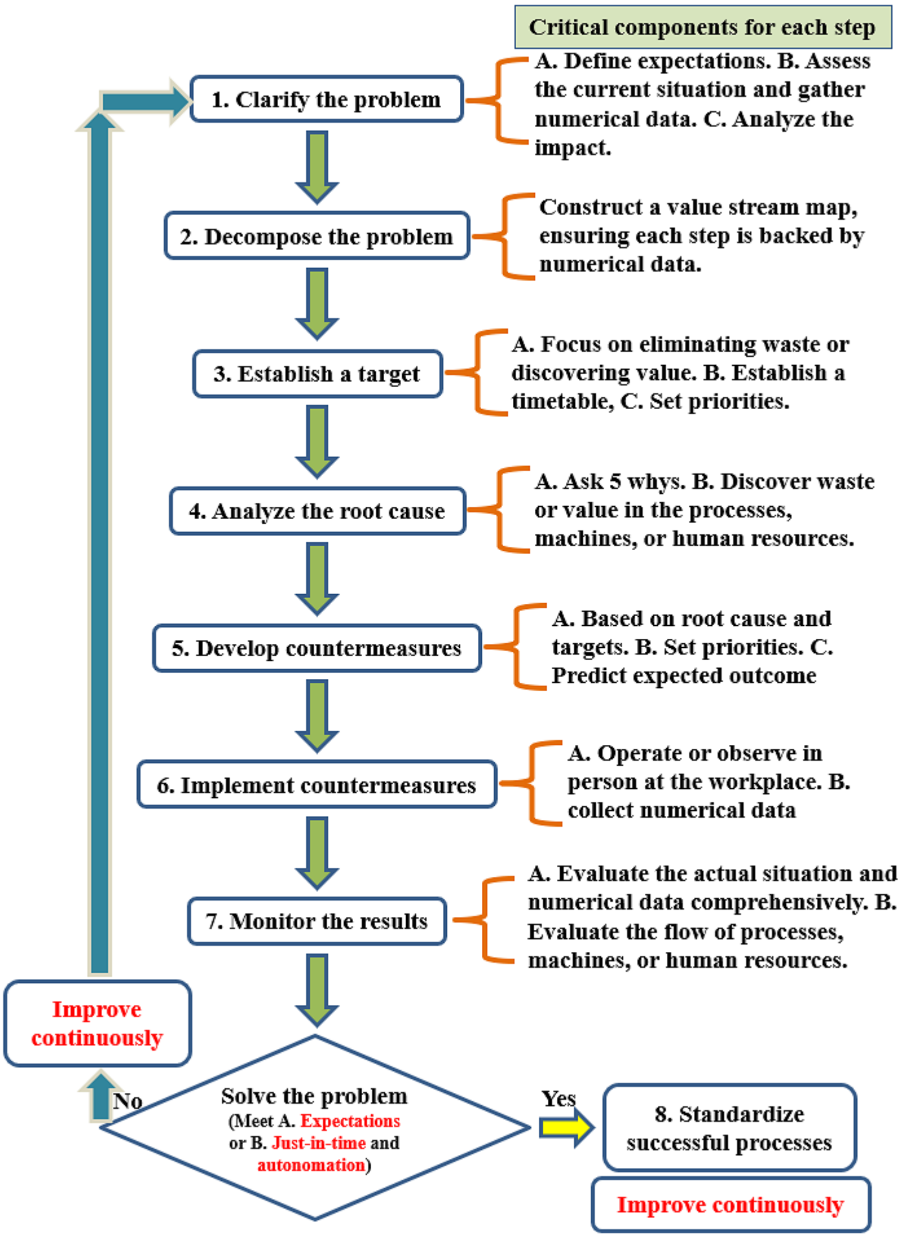

The TPS thinking referenced here stems from key insights by Mr. Taiichi Ohno, the founder of the TPS, as outlined in his 1988 book Toyota Production System [32]. Additional concepts are derived from Lean Thinking by Womack and Jones, who adopt the essence of the TPS, add their insights, and propose five principles of lean thinking. They also apply the concept of streams to embody each valuable step in accomplishing a task and use flow to run through all valuable steps, thus forming a value stream map [33]. These concepts provide essential frameworks for problem-solving and process improvement.

Ohno consistently emphasized his strong belief in the concept of "gemba" (on-site work). Even after joining Toyota's top management, he continued to spend most of his time at the workplace [32]. Therefore, it is no exaggeration to say that TPS is fundamentally about on-site management (**TPS Thinking 8**) [32-35]. According to Ohno's statements, work activities are classified based on their contribution to value into three categories: waste, non-value-added work (necessary but non-value-adding activities), and value-added work [32-33]. Waste refers to the use of resources without creating value. Non-value-added work, while necessary under current circumstances, does not generate value and is still considered a form of waste. Value-added work refers to activities that directly enhance the original value [32-33]. TPS thinking asserts that a product or service is valuable only if it meets the needs and expectations of the user or customer [32-35]. It is essential to observe the workplace carefully to clearly distinguish between value-adding activities and wasteful actions.

In the "Clarify the Problem" step, the current situation should be described using objectively observed facts, avoiding emotional responses or personal value judgments as much as possible. The numerical data collected throughout each step of the workflow should be based on objective and accurate measurements, in line with **TPS Thinking 1** [32-33]. This raises the question: which is more important, factual observations or numerical data? While Ohno valued numerical data, he believed that facts observed in the workplace were often more significant than the numbers alone [32]. From our experience, when numerical data and observed facts do not align, we take extra care to re-observe and verify the data.

From this study, it is clear that the data collected at the beginning allowed us to calculate averages or medians (see **Multimedia Appendix 1**). However, this data did not provide meaningful value in solving our problem. Since our goal was to quickly and immediately obtain available bed information, in the later stages of data

collection, we focused solely on gathering information that would help us achieve this goal as efficiently as possible, as shown by the seconds recorded in **Figures 3** and **9**. This data cannot be analyzed for statistical significance as in typical scientific experiments. Our research emphasizes factual events, with numerical data playing only a minor role. This approach aligns with Ohno's belief that directly observing events in the workplace and ensuring smooth processes is far more important than focusing exclusively on data analysis [32].

A VSM is constructed using accurate and objective facts about the current situation. This map is created by chronologically sequencing all valuable components of the work or process, following **TPS Thinking 2** [32-33,35-36]. In theory, every activity within the value stream should add value. However, in practice, even value-added activities may contain elements of waste or non-value-added work. Developing a detailed VSM to break down the problem is a critical step [32-33,35]. This article presents three levels of VSMs. The VSM for problem breakdown in **Figure 2** leads to the VSM in **Figure 3**, and the problem breakdown in **Figure 3** leads to the VSM in **Figure 5**. This step-by-step process of extracting value and eliminating waste is an ideal demonstration of problem decomposition.

When establishing a target, two approaches can be considered. The first approach involves eliminating all waste in the VSM, which is the most fundamental concept of TPS, as outlined in **TPS Thinking 3** [32-33]. The second approach focuses on directly extracting value from the VSM and using this to create a new VSM [32,36]. We were able to develop the Quick Isolation Bed Inquiry System only after adopting the second approach, following the failure of the first approach during the first and second cycles. It is crucial to emphasize that when setting a target, a strict deadline for completion must be enforced.

In the "analyzing root causes" step, the primary task is to use the "Five Whys" technique, asking "why" five times to uncover the root cause of the problem, with reference to **TPS Thinking 4** [32]. Commonly, asking "why" 2 to 3 times reveals the main cause, but it often requires 4 to 5 iterations to identify the true root cause. If the root cause is not addressed, the problem is likely to recur. Only by eliminating the root cause can the issue be fully resolved [32-35]. A cause can be considered the root cause if it consistently leads back to the observed problem and aligns with the collected facts and data. The inferred root causes in our case align with these principles.

According to **TPS Thinking 5** and **TPS Thinking 7**, the "Develop Countermeasures" step should prioritize solving process-related issues first, then machinery-related problems, and finally human-related concerns. Each countermeasure must clearly define the expected outcomes before implementation. During the "Implement Countermeasures" step, it is critical to return to the workplace to observe the actual conditions and simultaneously collect relevant numerical data. We have always adhered to this principle.

Meeting the expectations of users and customers can be considered as having achieved the goal, but only by fully attaining just-in-time and autonomation can it be called perfection [32-33].

Verify problem resolution

To evaluate whether a problem has been effectively resolved according to TPS standards, both subjective and objective criteria are applied. Subjectively, from a user or customer perspective, a problem is considered solved when the product or service meets their needs and expectations. However, the provider must ensure the solution also aligns with the core TPS principles: just-in-time and autonomation, the two pillars of TPS [32-33]. For a solution to be fully resolved by TPS standards, it must comply with these foundational principles.
