# Supplementary material for: Developing a Quick Isolation Bed Inquiry System During the COVID-19 Outbreak: User-Centered Design Approach Based on the Toyota Production System
Source: JMIR Form Res. 2025 Oct 17;9:e67152. doi: 10.2196/67152 (PMC12579300; doi:10.2196/67152)
Supplement: Multimedia Appendix 12 [file formative_v9i1e67152_app12.pdf]

The original Excel macro code developed for the Quick Isolation Bed Inquiry System has been adapted for compatibility with Microsoft Office 365.

```
Sub Covid19BedInqury()

Application.ScreenUpdating = False

'get this program     file name

thisProgramName = ThisWorkbook.Name

Dim mycopy As Integer

mymsg = "Press 1 ~~ Run Covid 19 Rapid Bed Inqury~~" & vbCr & vbCr & "Press Other keys to cancel the Program"

mytitle = "Covid 19 Rapid Bed Inqury"

mycopy = Application.InputBox(prompt:=mymsg, Title:=mytitle, Default:=1, Type:=1)

Heping = "**** branch"

'Change the following path for very computer (different file root)

'

rootPathOriginal = "E:\0 Lean- COVID 19 rapid bed query\English version 2024-8-

If mycopy = 1 Then      'decision: "Press Other keys to cancel the Program"



BedList = "Isolation ward bed numbers.xlsx"



NurseWriteFile = "Exported raw data.xls" ' Export raw data file saved as "Export raw data,xls"
```

```
'
'
'Delete all rows that patient's medical record number are not present in    the
“Export raw data” file
'
'
Workbooks.Open Filename:=rootPathOriginal & "\" & NurseWriteFile
'
'pick up run time
'
DayTime2 = Worksheets(1).Cells(2, 1).Value
DayTime1 = Mid(DayTime2, 12, 16) 'Chinese year
DayTime = (Year(DayTime1) + 1911) & "-" & Month(DayTime1) & "-" &
Day(DayTime1) 'change to Western year
'
'Delete all extra worksheet in the "Exported raw data.xls"
'
'
wc = Worksheets.Count
  Worksheets(wc).Activate

Application.DisplayAlerts = False
```

```
'

For I = Worksheets.Count To 2 Step -1

ActiveSheet.Delete



Next I

Application.DisplayAlerts = True

'count raw rows

'

NurseLastRow = ActiveSheet.UsedRange.Rows.Count

For hh = NurseLastRow To 4 Step -1

    If ActiveSheet.Cells(hh, "H").Value = "" Then    'delete all empty beds,
remaining having residence



       Rows(hh).Select



       Selection.Delete Shift:=xlUp



       End If

Next hh

'

'count new row number

'
```

```
NurseLastRowNew = ActiveSheet.UsedRange.Rows.Count

Dim occupiedBedNumber(500)


Dim gender(500)

For I = 4 To NurseLastRowNew 'read in bed numbers and genders and store them into varibles


occupiedBedNumber(I) = Range("A" & I).Value

gender(I) = Range("G" & I).Value


Next I


Application.DisplayAlerts = False


Workbooks(NurseWriteFile).Close 'close "Exported raw data.xls" file


Application.DisplayAlerts = True

'

'open Isolation ward bed numbers file

'

'

'read in data of Isolation ward bed numbers file into array
```

```
'
'

A6 = "HA6"
A7 = "HA7"
A8 = "HA8"
A9 = "HA9"
B6 = "HB6"
B7 = "HB7"
B8 = "HB8"
RCW = "HBR"
ICU = "HIC"

Workbooks.Open Filename:=rootPathOriginal & "\" & BedList

lastRow1 = ActiveSheet.UsedRange.Rows.Count 'BedList row count
'
'compare both files to delete occupied beds, ie. remainings are empty beds

For I = 4 To NurseLastRowNew
    For j = lastRow1 To 2 Step -1
```

```
            If Range("A" & j).Value = occupiedBedNumber(I) Then

              Rows(j).Select

              Selection.Delete Shift:=xlUp

             End If
          Next j

      Next I

      '
      'search female or male, find * ( valid twin beds in a room) and add a gender into the empty bed
      '
      '

        maleFemaleNo = ActiveSheet.UsedRange.Rows.Count
```

```
For uu = 2 To maleFemaleNo

    For j = NurseLastRowNew To 1 Step -1

        If Right(ActiveSheet.Cells(uu, "B").Value, 1) = "*" And
Left(ActiveSheet.Cells(uu, "A").Value, 5) = Left(occupiedBedNumber(j), 5) Then

            ActiveSheet.Cells(uu, "B").Value = ActiveSheet.Cells(uu,
"B").Value & gender(j)

        End If
```

```
    Next j

  Next uu

'
'
'count empty beds of each ward
'
'
Dim A9EmpBed(50)
Dim A8EmpBed(50)
Dim A7EmpBed(50)
Dim A6EmpBed(50)

A6EmpNo = 0

A7EmpNo = 0
```

```
A8EmpNo = 0

A9EmpNo = 0

ICUEmpNo = 0

CCount = maleFemaleNo

For ff = 2 To CCount

 If ActiveSheet.Cells(ff, "A").Value = "HICU17" Then

        ICU17Status = "occupied" ' count HICU17 if has a patient

  Else

        ICU17Status = "empty"
```

```
End If

If Left(ActiveSheet.Cells(ff, "A").Value, 3) = A9 Then

            A9EmpNo = A9EmpNo + 1

            A9EmpBed(A9EmpNo) = ActiveSheet.Cells(ff, "A") &
ActiveSheet.Cells(ff, "B")

End If

If Left(Worksheets(1).Cells(ff, "A").Value, 3) = A8 Then
```

```
A8EmpNo = A8EmpNo + 1

A8EmpBed(A8EmpNo) = Worksheets(1).Cells(ff, "A") & Worksheets(1).Cells(ff, "B")

End If

If Left(Worksheets(1).Cells(ff, "A").Value, 3) = A7 Then

A7EmpNo = A7EmpNo + 1

A7EmpBed(A7EmpNo) = Worksheets(1).Cells(ff, "A") & Worksheets(1).Cells(ff, "B")
```

```
  End If

  If Left(Worksheets(1).Cells(ff, "A").Value, 3) = A6 Then

                  A6EmpNo = A6EmpNo + 1

                  A6EmpBed(A6EmpNo) = Worksheets(1).Cells(ff, "A") & Worksheets(1).Cells(ff, "B")

  End If

Next ff
```

```
totalBedNumber = maleFemaleNo



'
'
'begin to compare
'
'
' Worksheets("原始表單").Activate


Dim StaffName(5) ' for store worksheet name


Worksheets(1).Activate


Worksheets(1).Name = "TotalEmptyBeds"        ' worksheets(1)
StaffName(1) = "TotalEmptyBeds"


Worksheets.Add after:=Worksheets("TotalEmptyBeds")
Worksheets(2).Name = "MobileFormOutput"
FinalOutput = "MobileFormOutput"
StaffName(2) = "MobileFormOutput"
```

```
Worksheets.Add after:=Worksheets(2)

Worksheets(3).Name = "ComputerFormOutput"

StaffName(3) = "ComputerFormOutput"




Worksheets.Add after:=Worksheets(3)

Worksheets(4).Name = "pdfFormOutput"

StaffName(4) = "pdfFormOutput"


Worksheets(1).Activate



'

'centerized all words

'

'

    Cells.Select

        With Selection

                .HorizontalAlignment = xlCenter
```

```
            .VerticalAlignment = xlCenter
            .WrapText = False
            .Orientation = 0
            .AddIndent = False
            .IndentLevel = 0
            .ShrinkToFit = False
            .ReadingOrder = xlContext
            .MergeCells = False
        End With

    Range("A1").Select

    '
    '
    ' "FinalOutput"
    '
    '

    Worksheets("MobileFormOutput").Activate
```

```
totalLast = 0

I = totalLast + 2

ActiveSheet.Cells(1, "A").Value = "xxxx branch"

ActiveSheet.Cells(1, "A").Font.Size = 13

ActiveSheet.Cells(1, "A").Font.Bold = True

ActiveSheet.Cells(1, 3).Value = "*Room for two"
ActiveSheet.Cells(1, 3).Font.Bold = True

ActiveSheet.Cells(1, 4).Value = "INS: mental instability(roommate)"
ActiveSheet.Cells(1, 4).Font.Bold = True
ActiveSheet.Cells(1, 4).Font.ColorIndex = 3
```

```
ActiveSheet.Cells(2, 3).Value = "Ask: call the nurse station"

ActiveSheet.Cells(2, 3).Font.Bold = True

ActiveSheet.Cells(2, 3).Font.ColorIndex = 3
```

```
ActiveSheet.Cells(1, "B").Value = DayTime

ActiveSheet.Cells(1, "B").Font.Bold = True

ActiveSheet.Cells(1, "B").Font.Size = 14
```

```
ActiveSheet.Cells(I, 1).Value = "Vacant bed No.:"

ActiveSheet.Cells(I, "A").Font.Size = 16
```

```
ActiveSheet.Cells(I, "A").Font.Bold = True

ActiveSheet.Cells(I, "A").Font.ColorIndex = 3

 I = I + 2

ActiveSheet.Cells(I, 1).Value = "A9:"

ActiveSheet.Cells(I, "A").Font.Size = 13

ActiveSheet.Cells(I, "A").Font.ColorIndex = 3

ActiveSheet.Cells(I, "A").Font.Bold = True
```

```
'
'
'break to 3 columns at each rows~ Mobile form
'
'

If A9EmpNo >= 1 Then

  xx = Round((A9EmpNo / 3) + 0.4)

  flagcount = 0

  For breakcount = 1 To xx
```

```
If breakcount < xx Then

For mm = 1 To 3

flagcount = flagcount + 1

hh = mm + 1

ActiveSheet.Cells(I, hh).Value = "Give~" &
A9EmpBed(flagcount)

ActiveSheet.Cells(I, hh).Font.Size = 16

Worksheets(FinalOutput).Cells(I, hh).Font.Bold = True
```

```
Next mm

l = l + 1

Worksheets(FinalOutput).Rows(l).Font.Size = 8

l = l + 1

End If

If breakcount = xx Then
```

```
For mm = 1 To (A9EmpNo - flagcount)

     flagcount = flagcount + 1

        hh = mm + 1

        Worksheets(FinalOutput).Cells(I, hh).Value = "Give~" &
A9EmpBed(flagcount)

        Worksheets(FinalOutput).Cells(I, hh).Font.Size = 16

        Worksheets(FinalOutput).Cells(I, hh).Font.Bold = True

 Next mm
```

```
    I = I + 1

      Worksheets(FinalOutput).Rows(I).Font.Size = 8

     I = I + 1

     End If

  Next breakcount

End If

If A9EmpNo <= 0 Then
```

```
    I = I + 1

        Worksheets(FinalOutput).Rows(I).Font.Size = 8

      I = I + 1

End If

Worksheets(FinalOutput).Cells(I, 1).Value = "A8:"

Worksheets(FinalOutput).Cells(I, "A").Font.Size = 13

      Worksheets(FinalOutput).Cells(I, "A").Font.ColorIndex = 3
```

```
        Worksheets(FinalOutput).Cells(I, "A").Font.Bold = True

'
'
'break to 3 columns at each rows
'
'
If A8EmpNo >= 1 Then

  xx = Round((A8EmpNo / 3) + 0.4)

  flagcount = 0

  For breakcount = 1 To xx
```

```
If breakcount < xx Then

        For mm = 1 To 3

        flagcount = flagcount + 1

        hh = mm + 1

        Worksheets(FinalOutput).Cells(I, hh).Value = "Give~" &
A8EmpBed(flagcount)

        Worksheets(FinalOutput).Cells(I, hh).Font.Size = 16

        Worksheets(FinalOutput).Cells(I, hh).Font.Bold = True

        Next mm
```

```
    l = l + 1

  Worksheets(FinalOutput).Rows(l).Font.Size = 8

l = l + 1

End If

If breakcount = xx Then

      For mm = 1 To (A8EmpNo - flagcount)
```

```
flagcount = flagcount + 1

hh = mm + 1

Worksheets(FinalOutput).Cells(I, hh).Value = "Give~" &
A8EmpBed(flagcount)

Worksheets(FinalOutput).Cells(I, hh).Font.Size = 16

Worksheets(FinalOutput).Cells(I, hh).Font.Bold = True

Next mm

I = I + 1
```

```
Worksheets(FinalOutput).Rows(I).Font.Size = 8

I = I + 1

End If

Next breakcount

End If

If A8EmpNo <= 0 Then
```

```
 I = I + 1

    Worksheets(FinalOutput).Rows(I).Font.Size = 8

   I = I + 1

End If

Worksheets(FinalOutput).Cells(I, 1).Value = "A7:"

Worksheets(FinalOutput).Cells(I, "A").Font.Size = 13

    Worksheets(FinalOutput).Cells(I, "A").Font.ColorIndex = 3

    Worksheets(FinalOutput).Cells(I, "A").Font.Bold = True
```

```
'
'
'break to 3 columns at each rows
'
'

  If A7EmpNo >= 1 Then

  xx = Round((A7EmpNo / 3) + 0.4)

  flagcount = 0

  For breakcount = 1 To xx

      If breakcount < xx Then
```

```
For mm = 1 To 3

flagcount = flagcount + 1

hh = mm + 1

Worksheets(FinalOutput).Cells(I, hh).Value = "Give~" &
A7EmpBed(flagcount)

Worksheets(FinalOutput).Cells(I, hh).Font.Size = 16

Worksheets(FinalOutput).Cells(I, hh).Font.Bold = True

Next mm
```

```
    l = l + 1

  Worksheets(FinalOutput).Rows(l).Font.Size = 8

l = l + 1

End If

If breakcount = xx Then

      For mm = 1 To (A7EmpNo - flagcount)
```

```
flagcount = flagcount + 1

hh = mm + 1

Worksheets(FinalOutput).Cells(I, hh).Value = "Give~" &
A7EmpBed(flagcount)

Worksheets(FinalOutput).Cells(I, hh).Font.Size = 16

Worksheets(FinalOutput).Cells(I, hh).Font.Bold = True

Next mm

I = I + 1
```

```
        Worksheets(FinalOutput).Rows(I).Font.Size = 8

       I = I + 1

       End If

   Next breakcount

End If

If A7EmpNo <= 0 Then
```

```
 I = I + 1

       Worksheets(FinalOutput).Rows(I).Font.Size = 8

     I = I + 1

End If

Worksheets(FinalOutput).Cells(I, 1).Value = "A6:"

Worksheets(FinalOutput).Cells(I, "A").Font.Size = 13

      Worksheets(FinalOutput).Cells(I, "A").Font.ColorIndex = 3

      Worksheets(FinalOutput).Cells(I, "A").Font.Bold = True
```

```
'
'
'break to 3 columns at each rows
'
'

If A6EmpNo >= 1 Then

  xx = Round((A6EmpNo / 3) + 0.4)

  flagcount = 0
```

```
For breakcount = 1 To xx

If breakcount < xx Then

For mm = 1 To 3

flagcount = flagcount + 1

hh = mm + 1

Worksheets(FinalOutput).Cells(I, hh).Value = "Give~" &
A6EmpBed(flagcount)

Worksheets(FinalOutput).Cells(I, hh).Font.Size = 16

Worksheets(FinalOutput).Cells(I, hh).Font.Bold = True
```

```
                    Next mm

        I = I + 1

    Worksheets(FinalOutput).Rows(I).Font.Size = 8

I = I + 1

End If
```

```
If breakcount = xx Then

    For mm = 1 To (A6EmpNo - flagcount)

        flagcount = flagcount + 1

            hh = mm + 1

            Worksheets(FinalOutput).Cells(I, hh).Value = "Give~" &
A6EmpBed(flagcount)

            Worksheets(FinalOutput).Cells(I, hh).Font.Size = 16

            Worksheets(FinalOutput).Cells(I, hh).Font.Bold = True
```

```
        Next mm

    l = l + 1

  Worksheets(FinalOutput).Rows(l).Font.Size = 8

  l = l + 1

  End If

Next breakcount
```

```
End If

If A6EmpNo <= 0 Then

  I = I + 1

     Worksheets(FinalOutput).Rows(I).Font.Size = 8

    I = I + 1

 End If
```

```
Worksheets(FinalOutput).Cells(I, 1).Value = "HICU17:"

Worksheets(FinalOutput).Cells(I, "A").Font.Size = 13

Worksheets(FinalOutput).Cells(I, "A").Font.ColorIndex = 3

Worksheets(FinalOutput).Cells(I, "A").Font.Bold = True


'
'
'HICU17
'
'


Worksheets(FinalOutput).Cells(I, 2).Value = ICU17Status




'
'
```

```
'

kk = 30

Worksheets(FinalOutput).Columns("A:A").ColumnWidth = 14
Worksheets(FinalOutput).Columns("B:B").ColumnWidth = kk
Worksheets(FinalOutput).Columns("C:C").ColumnWidth = kk
Worksheets(FinalOutput).Columns("D:D").ColumnWidth = kk

  kk = 60

  bbb = Worksheets(FinalOutput).UsedRange.Rows.Count

For gg = 4 To bbb + 1

Worksheets(FinalOutput).Rows(gg).RowHeight = kk
```

```
Next gg

 '
 'centrilized
 '

  Cells.Select
    With Selection
        .HorizontalAlignment = xlCenter
        .VerticalAlignment = xlCenter
        .WrapText = False
        .Orientation = 0
        .AddIndent = False
        .IndentLevel = 0
        .ShrinkToFit = False
        .ReadingOrder = xlContext
        .MergeCells = False
    End With
```

```
'
'hide '0" as empty ""
'

  'Cells.Select
  'Selection.NumberFormatLocal = "[=0]"""";G/通用格式"

'
'
'
'"ComputerFormOutput"
'
'
'

computerForm = "ComputerFormOutput"
```

```
Worksheets(computerForm).Activate

totalLast = 0

l = totalLast + 2

'
'
'row height and column width
'
'
'

kk = 60    ''' row height
```

```
Worksheets(computerForm).Columns("A:A").ColumnWidth = 18     'first columns
width

Worksheets(computerForm).Columns("B:P").ColumnWidth = 26 'others
columns width

Worksheets(computerForm).Cells(totalLast + 1, "A").Value = "xxxx branch"

Worksheets(computerForm).Cells(totalLast + 1, "A").Font.Size = 13

Worksheets(computerForm).Cells(totalLast + 1, "A").Font.Bold = True
```

```
Worksheets(computerForm).Cells(totalLast + 1, "B").Value = DayTime

Worksheets(computerForm).Cells(totalLast + 1, "B").Font.Bold = True

  Worksheets(computerForm).Cells(totalLast + 1, "B").Font.Size = 13

Worksheets(computerForm).Cells(1, 3).Value = "*Room for two"
Worksheets(computerForm).Cells(1, 3).Font.Bold = True

Worksheets(computerForm).Cells(1, 4).Value = "INS: mental
instability(roommate)"
Worksheets(computerForm).Cells(1, 4).Font.Bold = True
Worksheets(computerForm).Cells(1, 4).Font.ColorIndex = 3

Worksheets(computerForm).Cells(2, 3).Value = "Ask: call the nurse station"
Worksheets(computerForm).Cells(2, 3).Font.Bold = True
```

```
Worksheets(computerForm).Cells(2, 3).Font.ColorIndex = 3

Worksheets(computerForm).Cells(I, 1).Value = "Vacant bed No.:"

Worksheets(computerForm).Cells(I, "A").Font.Size = 14

Worksheets(computerForm).Cells(I, "A").Font.Bold = True

Worksheets(computerForm).Cells(I, "A").Font.ColorIndex = 3

I = I + 2

Worksheets(computerForm).Cells(I, 1).Value = "A9:"

Worksheets(computerForm).Cells(I, "A").Font.Size = 14

Worksheets(computerForm).Cells(I, "A").Font.ColorIndex = 3

Worksheets(computerForm).Cells(I, "A").Font.Bold = True
```

```
For mm = 1 To A9EmpNo

hh = mm + 1

Worksheets(computerForm).Cells(I, hh).Value = "Give~" & A9EmpBed(mm)

Worksheets(computerForm).Cells(I, hh).Font.Size = 16

Worksheets(computerForm).Cells(I, hh).Font.Bold = True

Next mm

'

'
```

```
'next row is empty and font=6,     '

'

'

'

l = l + 1

     Worksheets(computerForm).Rows(l).Font.Size = 8

     Worksheets(computerForm).RowHeight = kk

l = l + 1
```

```
Worksheets(computerForm).Cells(I, 1).Value = "A8:"

Worksheets(computerForm).Cells(I, "A").Font.Size = 14

    Worksheets(computerForm).Cells(I, "A").Font.ColorIndex = 3

    Worksheets(computerForm).Cells(I, "A").Font.Bold = True

For mm = 1 To A8EmpNo

hh = mm + 1

Worksheets(computerForm).Cells(I, hh).Value = "Give~" & A8EmpBed(mm)

Worksheets(computerForm).Cells(I, hh).Font.Size = 16
```

```
Worksheets(computerForm).Cells(I, hh).Font.Bold = True

Next mm

'
'
'next row is empty and font=6, autofit
'
'

I = I + 1

    Worksheets(computerForm).Rows(I).Font.Size = 8

    Worksheets(computerForm).Rows(I).RowHeight = kk
```

```
I = I + 1

Worksheets(computerForm).Cells(I, 1).Value = "A7:"

Worksheets(computerForm).Cells(I, "A").Font.Size = 14

Worksheets(computerForm).Cells(I, "A").Font.ColorIndex = 3

Worksheets(computerForm).Cells(I, "A").Font.Bold = True

  For mm = 1 To A7EmpNo

  hh = mm + 1

  Worksheets(computerForm).Cells(I, hh).Value = "Give~" & A7EmpBed(mm)
```

```
Worksheets(computerForm).Cells(I, hh).Font.Size = 16

Worksheets(computerForm).Cells(I, hh).Font.Bold = True

Next mm

'
'
'next row is empty and font=6, autofit
'
'

I = I + 1

    Worksheets(computerForm).Rows(I).Font.Size = 8
```

```
Worksheets(computerForm).Rows(I).RowHeight = kk

I = I + 1

Worksheets(computerForm).Cells(I, 1).Value = "A6:"

Worksheets(computerForm).Cells(I, "A").Font.Size = 14

Worksheets(computerForm).Cells(I, "A").Font.ColorIndex = 3

Worksheets(computerForm).Cells(I, "A").Font.Bold = True

For mm = 1 To A6EmpNo

hh = mm + 1
```

```
Worksheets(computerForm).Cells(I, hh).Value = "Give~" & A6EmpBed(mm)

Worksheets(computerForm).Cells(I, hh).Font.Size = 16

Worksheets(computerForm).Cells(I, hh).Font.Bold = True

Next mm

'
'
'next row is empty and font=6, autofit
'
'
```

```
 I = I + 1

       Worksheets(computerForm).Rows(I).Font.Size = 8

       Worksheets(computerForm).Rows(I).RowHeight = kk

I = I + 1

 Worksheets(computerForm).Cells(I, 1).Value = "HICU17:"

 Worksheets(computerForm).Cells(I, "A").Font.Size = 13

       Worksheets(computerForm).Cells(I, "A").Font.ColorIndex = 3
```

```
    Worksheets(computerForm).Cells(I, "A").Font.Bold = True

'
'
'HICU17
'
'

Worksheets(computerForm).Cells(I, 2).Value = ICU17Status

I = I + 1

    Worksheets(computerForm).Rows(I).Font.Size = 8
```

```
    Worksheets(computerForm).Rows(I).RowHeight = kk




'
'centrilized and grids
'


    Cells.Select
    With Selection
        .HorizontalAlignment = xlCenter
        .VerticalAlignment = xlCenter
        .WrapText = False
        .Orientation = 0
        .AddIndent = False
        .IndentLevel = 0
        .ShrinkToFit = False
        .ReadingOrder = xlContext
        .MergeCells = False
    End With
```

```
Rows("4:15").Select
Selection.Borders(xlDiagonalDown).LineStyle = xlNone
Selection.Borders(xlDiagonalUp).LineStyle = xlNone
With Selection.Borders(xlEdgeLeft)
    .LineStyle = xlContinuous
    .ColorIndex = xlAutomatic
    .TintAndShade = 0
    .Weight = xlThin
End With
With Selection.Borders(xlEdgeTop)
    .LineStyle = xlContinuous
    .ColorIndex = xlAutomatic
    .TintAndShade = 0
    .Weight = xlThin
End With
With Selection.Borders(xlEdgeBottom)
    .LineStyle = xlContinuous
    .ColorIndex = xlAutomatic
    .TintAndShade = 0
    .Weight = xlThin
End With
With Selection.Borders(xlEdgeRight)
```

```
            .LineStyle = xlContinuous
            .ColorIndex = xlAutomatic
            .TintAndShade = 0
            .Weight = xlThin
        End With
        With Selection.Borders(xlInsideVertical)
            .LineStyle = xlContinuous
            .ColorIndex = xlAutomatic
            .TintAndShade = 0
            .Weight = xlThin
        End With
        With Selection.Borders(xlInsideHorizontal)
            .LineStyle = xlContinuous
            .ColorIndex = xlAutomatic
            .TintAndShade = 0
            .Weight = xlThin
        End With


    '
    'hide '0" as empty ""
    '
```

```
'Cells.Select

'Selection.NumberFormatLocal = "[=0]"""";G/通用格式"

'

'

'

'

'        "pdfFormOutput" for publish

'

'

'

'

broadCastForm = "pdfFormOutput"

totalLast = 0
```

```
l = totalLast + 2

Worksheets(broadCastForm).Cells(1, "A").Value = "xxxx branch"

Worksheets(broadCastForm).Cells(1, "A").Font.Size = 13

Worksheets(broadCastForm).Cells(1, "A").Font.Bold = True

Worksheets(broadCastForm).Cells(1, 3).Value = "*Room for two"
Worksheets(broadCastForm).Cells(1, 3).Font.Bold = True

Worksheets(broadCastForm).Cells(1, 4).Value = "INS: mental
instability(roommate)"
Worksheets(broadCastForm).Cells(1, 4).Font.Bold = True
Worksheets(broadCastForm).Cells(1, 4).Font.ColorIndex = 3
```

```
Worksheets(broadCastForm).Cells(2, 3).Value = "Ask: call the nurse station"

Worksheets(broadCastForm).Cells(2, 3).Font.Bold = True

Worksheets(broadCastForm).Cells(2, 3).Font.ColorIndex = 3



Worksheets(broadCastForm).Cells(1, "B").Value = DayTime


Worksheets(broadCastForm).Cells(1, "B").Font.Bold = True


Worksheets(broadCastForm).Cells(1, "B").Font.Size = 14



Worksheets(broadCastForm).Cells(3, 1).Value = "Vacant bed No.:"


Worksheets(broadCastForm).Cells(3, "A").Font.Size = 16


Worksheets(broadCastForm).Cells(3, "A").Font.Bold = True


Worksheets(broadCastForm).Cells(3, "A").Font.ColorIndex = 3
```

```
 I = I + 2

Worksheets(broadCastForm).Cells(I, 1).Value = "A9:Empty " & A9EmpNo & " bed(s)"

Worksheets(broadCastForm).Cells(I, "A").Font.Size = 13

Worksheets(broadCastForm).Cells(I, "A").Font.ColorIndex = 3

Worksheets(broadCastForm).Cells(I, "A").Font.Bold = True

'
'
'break to 3 columns at each rows
'
'
```

```
If A9EmpNo >= 1 Then

  xx = Round((A9EmpNo / 3) + 0.4)

  flagcount = 0

  For breakcount = 1 To xx

       If breakcount < xx Then

                       For mm = 1 To 3

                       flagcount = flagcount + 1
```

```
hh = mm + 1

Worksheets(broadCastForm).Cells(l, hh).Value =
A9EmpBed(flagcount)

Worksheets(broadCastForm).Cells(l, hh).Font.Size = 16

Worksheets(broadCastForm).Cells(l, hh).Font.Bold = True

Next mm

l = l + 1

End If
```

```
If breakcount = xx Then

    For mm = 1 To (A9EmpNo - flagcount)

        flagcount = flagcount + 1

            hh = mm + 1

            Worksheets(broadCastForm).Cells(I, hh).Value =
A9EmpBed(flagcount)

            Worksheets(broadCastForm).Cells(I, hh).Font.Size = 16
```

```
Worksheets(broadCastForm).Cells(I, hh).Font.Bold = True

Next mm

I = I + 1

End If

Next breakcount

End If
```

```
If A9EmpNo <= 0 Then

    I = I + 1

  End If

  Worksheets(broadCastForm).Cells(I, 1).Value = "A8:Empty " & A8EmpNo & "
bed(s)"

  Worksheets(broadCastForm).Cells(I, "A").Font.Size = 13
```

```
  Worksheets(broadCastForm).Cells(I, "A").Font.ColorIndex = 3

  Worksheets(broadCastForm).Cells(I, "A").Font.Bold = True


'
'
'break to 3 columns at each rows
'
'
If A8EmpNo >= 1 Then

  xx = Round((A8EmpNo / 3) + 0.4)

  flagcount = 0

  For breakcount = 1 To xx
```

```
If breakcount < xx Then

For mm = 1 To 3

flagcount = flagcount + 1

hh = mm + 1

Worksheets(broadCastForm).Cells(I, hh).Value =
A8EmpBed(flagcount)

Worksheets(broadCastForm).Cells(I, hh).Font.Size = 16

Worksheets(broadCastForm).Cells(I, hh).Font.Bold = True

Next mm
```

```
 l = l + 1
End If
If breakcount = xx Then
    For mm = 1 To (A8EmpNo - flagcount)
        flagcount = flagcount + 1
            hh = mm + 1
```

```
                    Worksheets(broadCastForm).Cells(I, hh).Value =
A8EmpBed(flagcount)

                    Worksheets(broadCastForm).Cells(I, hh).Font.Size = 16

                    Worksheets(broadCastForm).Cells(I, hh).Font.Bold = True

            Next mm

          I = I + 1

        End If

    Next breakcount
```

```
End If

If A8EmpNo <= 0 Then

  I = I + 1

End If

Worksheets(broadCastForm).Cells(I, 1).Value = "A7:Empty " & A7EmpNo & " bed(s)"

Worksheets(broadCastForm).Cells(I, "A").Font.Size = 13
```

```
  Worksheets(broadCastForm).Cells(I, "A").Font.ColorIndex = 3

  Worksheets(broadCastForm).Cells(I, "A").Font.Bold = True

'
'
'break to 3 columns at each rows
'
'

  If A7EmpNo >= 1 Then

  xx = Round((A7EmpNo / 3) + 0.4)

  flagcount = 0

  For breakcount = 1 To xx
```

```
If breakcount < xx Then

        For mm = 1 To 3

        flagcount = flagcount + 1

        hh = mm + 1

        Worksheets(broadCastForm).Cells(I, hh).Value =
A7EmpBed(flagcount)

        Worksheets(broadCastForm).Cells(I, hh).Font.Size = 16

        Worksheets(broadCastForm).Cells(I, hh).Font.Bold = True
```

```
            Next mm

    I = I + 1

End If

If breakcount = xx Then

    For mm = 1 To (A7EmpNo - flagcount)

        flagcount = flagcount + 1
```

```
hh = mm + 1

Worksheets(broadCastForm).Cells(I, hh).Value =
A7EmpBed(flagcount)

Worksheets(broadCastForm).Cells(I, hh).Font.Size = 16

Worksheets(broadCastForm).Cells(I, hh).Font.Bold = True

Next mm

I = I + 1
```

```
        End If

   Next breakcount

End If

If A7EmpNo <= 0 Then

    I = I + 1

  End If
```

```
'
 Worksheets(broadCastForm).Cells(I, 1).Value = "A6:Empty " & A6EmpNo & "
bed(s)"
 Worksheets(broadCastForm).Cells(I, "A").Font.Size = 13
 Worksheets(broadCastForm).Cells(I, "A").Font.ColorIndex = 3
 Worksheets(broadCastForm).Cells(I, "A").Font.Bold = True
'
'
'break to 3 columns at each rows
'
```

```
'

If A6EmpNo >= 1 Then

  xx = Round((A6EmpNo / 3) + 0.4)

  flagcount = 0

  For breakcount = 1 To xx

        If breakcount < xx Then

                For mm = 1 To 3
```

```
flagcount = flagcount + 1

hh = mm + 1

Worksheets(broadCastForm).Cells(I, hh).Value =
A6EmpBed(flagcount)

Worksheets(broadCastForm).Cells(I, hh).Font.Size = 16

Worksheets(broadCastForm).Cells(I, hh).Font.Bold = True

Next mm

I = I + 1
```

```
End If

If breakcount = xx Then

    For mm = 1 To (A6EmpNo - flagcount)

        flagcount = flagcount + 1

            hh = mm + 1

            Worksheets(broadCastForm).Cells(I, hh).Value =
A6EmpBed(flagcount)
```

```
                Worksheets(broadCastForm).Cells(I, hh).Font.Size = 16

                Worksheets(broadCastForm).Cells(I, hh).Font.Bold = True

            Next mm

          I = I + 1

        End If

      Next breakcount

    End If
```

```
If A6EmpNo <= 0 Then

  I = I + 1

 End If

 Worksheets(broadCastForm).Cells(I, 1).Value = "HICU17:"

 Worksheets(broadCastForm).Cells(I, "A").Font.Size = 13

 Worksheets(broadCastForm).Cells(I, "A").Font.ColorIndex = 3

 Worksheets(broadCastForm).Cells(I, "A").Font.Bold = True
```

```
 '
 '
 'HICU17
 '
 '

 Worksheets(broadCastForm).Cells(I, 2).Value = ICU17Status

kk = 20

Worksheets(broadCastForm).Columns("A:A").ColumnWidth = 14
Worksheets(broadCastForm).Columns("B:B").ColumnWidth = kk
Worksheets(broadCastForm).Columns("C:C").ColumnWidth = kk
Worksheets(broadCastForm).Columns("D:D").ColumnWidth = kk

kk = 30
```

```
bbb = Worksheets(broadCastForm).UsedRange.Rows.Count

For gg = 4 To bbb + 1

Worksheets(broadCastForm).Rows(gg).RowHeight = kk

Next gg

'
'=================================================================
==================================================
'
nowTime = Now()

addDay = Year(nowTime) & "-" & Month(nowTime) & "-" & Day(nowTime)
```

```
'nowTimeSave = "-" & addDay & "日-執行程式-" & Hour(nowTime) & "點" & Minute(nowTime) & "分" & Second(nowTime) & "秒"

nowTimeSave = "-" & addDay & "-Execute Program at " & Hour(nowTime) & "h-" & Minute(nowTime) & "m-" & Second(nowTime) & "s"

'"pdfFormOutput--2024-6-12 - Execute Program - 13:25:39"
'==================================================================================================================================
' MsgBox "所有工作表都已存成獨立的檔案！", vbInformation
'

AA0102 = "All Information" & "-" & nowTimeSave



  source_window_name = ActiveWindow.Caption

'MsgBox "source_window_name=" & source_window_name

source_path_name = ActiveWorkbook.Path
'先把現在準備分割的原始檔案的視窗名稱及路徑記錄下來
```

```
target_path = source_path_name & "\" & Left(source_window_name, Len(source_window_name) - 5) & nowTimeSave

'MsgBox "target_path=" & target_path

MkDir target_path

Application.DisplayAlerts = False

ActiveWorkbook.SaveAs target_path & "\" & AA0102

Application.DisplayAlerts = True

'
'seperate to different workbooks
'

For tt = 1 To 4

    Dim sourceWs As Worksheet
```

```
Dim newWb As Workbook
Dim newWs As Worksheet

' 創建新的工作簿
Set newWb = Workbooks.Add
Set newWs = newWb.Sheets(1)
newWs.Name = StaffName(tt)

' 檢查「A workbook.xlsm」是否已經打開
' If Not IsWorkbookOpen(AA0102) Then
    ' MsgBox AA0102 & "未開啟"
    ' Exit Sub
'End If

' 參考「A workbook.xlsm」的「Aa」工作表
Set sourceWs = Workbooks(AA0102).Worksheets(StaffName(tt))

' 複製工作表內容到新的工作簿的「林中」工作表
sourceWs.Cells.Copy newWs.Cells

    Application.DisplayAlerts = False
```

```
ActiveWorkbook.SaveAs target_path & "\" & StaffName(tt) & "-" &
nowTimeSave

Application.DisplayAlerts = True

ActiveWorkbook.Close

Next tt

'
'
'directly save as pdf file
'
'

broadCastForm = broadCastForm & "-" & nowTimeSave 'pdf

Workbooks.Open Filename:=target_path & "\" & broadCastForm
```

```
  ActiveWorkbook.ExportAsFixedFormat Type:=xlTypePDF,
Filename:=target_path & "\" & broadCastForm & ".pdf"

        ActiveWorkbook.FollowHyperlink target_path & "\" & broadCastForm &
".pdf"

'Workbooks.Open Filename:=target_path & "\" & StaffName(3) & "-" &
nowTimeSave

'Workbooks.Open Filename:=target_path & "\" & StaffName(2) & "-" &
nowTimeSave

        Application.DisplayAlerts = False

        Workbooks(broadCastForm).Close
```

```
        Workbooks(thisProgramName).Close

        Application.DisplayAlerts = True

    MsgBox "所有工作表都已保存到 " & target_path, vbInformation, "完成"

Range("A1").Select

Else
```

```
    MsgBox "其他鍵~~~取消"

End If

End Sub
```
